# Bee pollen peptides as potent tyrosinase inhibitors with anti-melanogenesis effects in murine b16f10 melanoma cells and zebrafish embryos

**DOI:** 10.1038/s41598-024-81495-8

**Published:** 2024-12-28

**Authors:** Papassara Sangtanoo, Piroonporn Srimongkol, Tanatorn Saisavoey, Songchan Puthong, Anumart Buakeaw, Rutairat Suttisuwan, Marisa Jatupornpipat, Wittaya Pimtong, Onrapak Reamtong, Aphichart Karnchanatat

**Affiliations:** 1 Center of Excellence in Bioconversion and Bioseparation for Platform Chemical Production, Institute of Biotechnology and Genetic Engineering, Chulalongkorn University, 254 Phayathai Road, Pathumwan, Bangkok, 10330 Thailand; 2 Biodiversity and Sustainable Utilization Research Unit, Department of Biology, Faculty of Science and Technology, Rajamangala University of Technology Krungthep, 2 Nang linchi Road, Sathorn, Bangkok, 10120 Thailand; 3 Department of Biology, Faculty of Science, King Mongkut’s Institute of Technology, Ladkrabang, Chalongkrung Road, Ladkrabang, Bangkok, 10520 Thailand; 4 Nano Environmental and Health Safety Research Team, National Science and Technology Development Agency (NSTDA), 111 Thailand Science Park, Phahonyothin Road, Khlong Nueng, Khlong Luang, 12120 Pathum Thani Thailand; 5 Department of Molecular Tropical Medicine and Genetics, Faculty of Tropical Medicine, Mahidol University, 420/6 Ratchawithi Road, Ratchathewi, Bangkok, 10400 Thailand

**Keywords:** Bee pollen, Protein hydrolysate, Tyrosinase inhibitory peptides, Melanogenesis, B16F10 mouse melanoma cells, Zebrafish, Biochemistry, Biotechnology

## Abstract

One important functional food ingredient today, valued for its health properties and ability to prevent disease, is bee pollen, which comprises a combination of nectar, pollen from plants, and the secretions of bees. In this research, the tyrosinase (TYR) inhibiting abilities of the peptides derived from bee pollen protein hydrolysates are investigated. Various proteases were utilized to generate these peptides, followed by testing at different concentrations. Tyrosinase inhibition activity was detected in all cases, while the hydrolysate drawn from 5.0% w/v neutrase exhibited the best IC_50_ value and was thus investigated further via ultrafiltration to separate the active fractions. The highest potential for tyrosinase inhibition was recorded for the fractions below 0.65 kDa. Subsequent purification steps via SEC and RP-HPLC led to the identification of the VDGYPAAGY (named VY-9) peptide via LC-Q-TOF-MS/MS in fraction F_1–2_, known for its non-toxic and hydrophobic characteristics albeit poor water solubility. The synthesized VY-9 peptide demonstrated competitive inhibition, with IC_50_ values of 0.55 ± 0.03 µM for mono-phenolase and 2.54 ± 0.06 µM for di-phenolase activities, as confirmed by molecular docking analysis revealing dominant hydrogen bond interactions with TYR. Effective concentrations of 0.2–1.6 µM of VY-9 showed negligible cytotoxicity in B16F10 cells. Melanin synthesis suppression was examined via qRT-PCR, and western blot in MITF, TYR, TRP-1, and TRP-2. Cell death in zebrafish embryos was evaluated in vivo using a toxicity assay which revealed no significant influence from VY-9, while anti-melanogenic effects were observed when the concentration was 4 µM, suggesting bee pollen-derived peptides’ potential in cosmetic and pharmaceutical depigmentation applications.

**Supplementary Information:**

The online version contains supplementary material available at 10.1038/s41598-024-81495-8.

## Introduction

Quality of life can be adversely affected by cosmetic skin problems, and one common remedy involves lightening through the use of either synthetic or natural substances which lighten the skin tone, or in some cases even the skin complexion by lowering the melanin levels in the skin. Accordingly, skin lightening can be used to address problems with freckles, discolored skin, or scars resulting from acne. Skin lightening products typically promise perfect glowing skin, and have long been popular with women, and also today with men^1–3^. In Asia there is significant demand for products to whiten the skin. Since Asian skin tends to be naturally more hydrated, problems of hyperpigmentation or hypopigmentation are more frequently observed. As Asian people age, they are less likely to wrinkle but more likely to exhibit an uneven skin tone. Furthermore, many Asians want to look more western, so try to whiten their skin^2^. In mammals, melanin is the predominant skin pigment, and it is formed from melanocytes as tyrosine undergoes enzymatic oxidation. Melanin offers protection against ultraviolet (UV) irradiation, DNA damage, and oxidative stress, all of which are harmful to the skin. Protection against UV irradiation also helps in preventing skin cancer. Melanin also has the negative effect, however, of causing mottled or sunburned skin, so substances capable of blocking the synthesis of melanin are likely to be effective as cosmetic products to whiten the skin. The synthesis of melanin takes place in melanosomes whereupon it moves to the nearby epidermal keratinocytes. There are a number of melanocyte-specific enzymes such as tyrosinase (TYR), TYR-related protein 1 (TRP-1), and TYR-related protein 2 (TRP-2) which serve to regulate the process of melanogenesis^4–6^.

One role of TYR, which is a binuclear copper enzyme, is to regulate melanogenesis. It acts as a catalyst for the hydroxylation of tyrosine and the oxidation of 3, 4-dihydroxyphenylalanine (L-DOPA) to o-dopaquinone which are the rate limiting reactions which occur during the synthesis of melanin. Skin color is dependent upon the level of melanin synthesis as well as the type of melanin and the way it is distributed through the keratinocytes. Achieving effective inhibition of TYR with minimal adverse side effects has remained a longstanding challenge within the realms of dermatological and cosmetological sciences. Despite the existence of various potent TYR inhibitors, such as sulfite or kojic acid, their utilization is limited. However, these substances are not utilized due to their high degree of toxicity towards cells and instability in the presence of water or oxygen^7–9^. It is vital that inhibitors used in the food or cosmetics sectors meet the highest safety standards, so while various synthetic and natural TYR inhibitors are known, they are largely unsuitable for use as skin whiteners due to their cytotoxicity, poor solubility, or lack of suitability in terms of cutaneous absorption. Some of the TYR inhibitors which have found application in skin whitening products have been shown to be carcinogenic; the WHO has advised that some skin lightening agents can lead to cancer, and notes that mercury, which is a harmful toxin, is sometimes used in skin whitening products in order to inhibit the production of melanin^10,11^. In this context, natural or organic products are likely to become increasingly popular, so researchers have sought to focus on natural peptides since they are relatively safe and can be very effective. Accordingly, various studies have been carried out to examine TYR inhibitor peptides derived from synthetic peptides as well as proteins and protein hydrolysates to determine their mechanisms and efficacy. Natural compounds have drawn the greatest attention since they are perceived to lack undesirable side effects. In addition, they offer the advantages of peptides with protein mimicking and high biocompatibility, and therefore have potential to serve as active ingredients in newly developed products for the cosmetic and pharmaceutical sectors^12,13^. Accordingly, it appears that TYR activity can be inhibited by peptides and proteins from common sources including milk^14^, split gill mushrooms^15^, honey^16^, silk^17^, feather^18^, spotted babylon^19^, and the jellyfish^20^.

Bee pollen is produced in hives from nectar, the saliva of the bees, and flower pollen grains. It can be safely harvested from the hive by beekeepers. It is rich in nutrition, providing lipids, carbs, proteins, and dietary fibers along with minerals, vitamins, and both phenolic and volatile compounds. Around 200 different substances have been found in the pollen from various plant species, but bee pollen remains notable due to its biologically active components. From a nutritional perspective it can be classified as a functional food, which has invited studies to examine its potential in both the medical and food industries^21,22^. In recent years, studies have investigated the possibility of improving upon the capabilities of bee pollen via the process of enzymatic hydrolysis. The bee pollen protein hydrolysate (BPPH) can undergo hydrolysis to create natural bioactive peptides, as reported by Saisavoey et al.^23^. who showed that the enzymatic hydrolysates derived from bee pollen are effective in NO scavenging, while the neutrase hydrolysate can produce peptides which have anti-inflammatory properties. The production of NO is inhibited by the smallest peptide fraction (< 0.65 kDa), which can also counteract the LPS-induced expression of IL-6, COX-2, iNOS, and TNF-α transcripts in RAW264.7 macrophage cells, confirming that enzymatic hydrolysis can lead to better anti-inflammatory activity. Saisavoey et al.^24^. reported that bee pollen treated with alcalase could serve as an antioxidant, while antiproliferation activity was exhibited towards lung cancer cells (ChaGo-K1) by the BPPH, thus promoting apoptosis. The production of TIPs (tyrosinase inhibitory peptides) from BPPH has not, however, been widely reported. It is claimed that these bioactivities result from the greater variety and overall numbers of bioactive peptides which can be generated through enzymatic hydrolysis. The role of bioactive peptides in human nutrition has attracted widespread interest, but this study focuses on bee pollen bioactive peptides derived from enzymatic hydrolysis and investigates their ability to inhibit melanogenesis. We took the view that bee pollen might be a potential source of TIPs with the capacity to inhibit the expression of TYR in B16F10 mouse melanoma cells through disruption of the melanogenesis signaling cascades. In addition, in vivo testing of the ability of peptides to inhibit melanin generation was performed in zebrafish embryos. It can thus be concluded that bee pollen may be a source of valuable bioactive peptides which can find useful applications in the cosmetic sector as an important ingredient.

## Results and discussion

### Enrichment of bee pollen peptides treated with different proteases

TIPs are now widely prepared via enzymatic hydrolysis, due to the ease of controlling the process and the absence of extreme reaction conditions. There are many commercially produced enzymes which are available to support TIP production, among which are trypsin, chymotrypsin, flavourzyme, alkaline protease, neutral protease, papain, and so forth. It is understood that different enzymes can differ in their hydrolytic impact upon a given material because the binding specificity between substrate and enzymes will vary. However, in the case of the enzymolytic effect of an enzyme, the same enzyme will produce different results on different materials^12^. In this study, the enzymes under investigation included neutrase, alcalase, flavourzyme, papain, and pepsin-pancresin. Hydrolysis of the bee pollen powder was conducted with various proteases at three specific concentrations before comparisons were drawn to consider the TYR inhibition of the bee pollen hydrolysates, with findings shown in Table 1. The lowest IC_50_ values for the mono-phenolase activity (50.01 ± 1.15 µg/mL) and di-phenolase activity (39.93 ± 0.60 µg/mL) were recorded for the protein hydrolysate which was prepared using 5% (w/v) neutrase. The hydrolysates comprise a number of peptides, and thus differ from kojic acid, which is a single molecule and a very potent standard TYR inhibitor. For this reason, the true concentration of the active compound is not as high as that stated for the IC_50_ value. This study therefore places emphasis upon those hydrolysates which offer the greatest enzyme inhibition^7^. A similar example can be found in the work of Zhao et al.^25^. whose hydrolysis of the “Fengdan” peony (*Paeonia ostii*) seed meal protein with neutrase (at 1 mg/mL) revealed hydrolysates achieving 59.7% TYR inhibition. Before carrying out the process of enzymatic hydrolysis it is essential to select the appropriate proteases and raw materials, because the protein amino acid compositions vary and this leads to different sized peptides being released, which offer different levels of bioactivity.

**Table 1:** The IC_50_ values of the TYR inhibitory activity of BPPH derived from different enzymes.

Enzyme % (w/v)	Neutrase	Flavourzyme	Alcalase	Pepsin/Pancreatin	Papain
	L-Tyrosine (mono-phenolase activity) IC_50_ (µg/mL)				
1.0	139.90 ± 1.74^e^	293.63 ± 2.05^i^	243.00 ± 5.50^h^	69.76 ± 0.35^b^	224.30 ± 10.10^g^
2.5	136.60 ± 9.18^e^	98.32 ± 0.19^c^	223.97 ± 7.37^g^	56.29 ± 0.44^a^	189.90 ± 4.19^f^
5.0	50.01 ± 1.15^a^	53.69 ± 2.84^a^	117.67 ± 2.16^d^	51.57 ± 0.98^a^	65.89 ± 5.92^b^
	L-DOPA (di-phenolase activity) IC_50_ (µg/mL)				
1.0	180.20 ± 4.92^f^	286.30 ± 10.77^i^	174.70 ± 8.29^f^	63.80 ± 159^c^	275.93 ± 8.62^h^
2.5	118.43 ± 6.10^d^	48.19 ± 0.68^ab^	158.20 ± 8.97^e^	56.82 ± 2.82^bc^	267.10 ± 4.69^h^
5.0	39.93 ± 0.60^a^	43.20 ± 5.78^a^	65.54 ± 8.78^c^	42.80 ± 1.15^a^	248.40 ± 3.20^g^

Data are expressed in the form of mean ± standard error and the results are generated in triplicate. ^a-h^Those values with different letters appearing in the same row show significant differences (*P* < 0.05).

The IC_50_ values of kojic acid as a positive control were 16.50 ± 1.95 µg/mL for mono-phenolase and 118.23 ± 3.19 µg/mL for di-phenolase.

Those proteins offering strong TYR inhibition typically contain high levels of hydrophobic amino acids, such as Trp, Phe, Gly, Val, Leu, Ile, Ala, Pro, and Met, and also aromatic amino acids including Tyr, Trp, and Phe. Neutrase, which can be described as a type of neutral protease has a tendency to hydrolyze proteins to generate peptides which have hydrophobic amino acids including Tyr, Trp, or Phe as *C*-terminals^26^. Accordingly, neutrase could serve as an appropriate protease for the preparation of high-activity TIPs. Reactions take place between hydrogen donor hydrophobic amino acids and the various residues, free radicals, or metal ions, while the aromatic amino acids possess conjugated planar rings, which are able to absorb ultraviolet rays while also engaging in π-π interactions with the copper ions of TYR. The oxidative activity of TYR tends to be disrupted by the conjugation with Cu^2+^, which in turn limits melanin synthesis^12^. To summarize, the current approach to obtain TIPs involves the screening of enzyme species followed by optimization of the enzymolytic process along with efficacious purification. This study employed neutrase in sequence since this was the approach which provided the highest levels of TYR inhibition. These findings were then taken forward to guide the ultrafiltration (UF) stage.

### UF of the prepared BPPH

Having obtained the enzymatic hydrolysates from protein fractions and peptides, it is necessary to perform further filtration in order to separate these peptides prior to carrying out additional experimentation. The peptides must be purified isolated from other substances before it is possible to determine their abilities in melanogenesis inhibition in cell culture or to assess their anti-TYR properties through in vitro assay. This is because impurities could affect the outcomes of these analyses. Most research into bioactive peptides makes use of membrane fractionation techniques to commence the analysis. The purification stage begins by filtering the protein hydrolysates and peptides using membranes which are able to separate the fractions by molecular size. The food sector normally requires UF in which the sub-fractions fall within a range of 1 to 100 kDa for molecular size^27^. The initial screening for bioactivity can then be carried out by comparing the activity levels of the different sub-fractions. The most promising protein hydrolysate can be separated by membranes to form different peptide sizes, typically of MW > 10 kDa, MW 5–10 kDa, MW 3–5 kDa, MW 3-0.65, and MW < 0.65 kDa. Assays can then be conducted for in vitro TYR inhibition to determine the capacity to serve as TYR inhibitors in mono-phenolase and di-phenolase contexts, as explained in the section describing the experimental procedures. Table 2 presents the findings. The lowest IC_50_ values for both mono-phenolase activity (1.08 ± 0.50 µg/mL) and di-phenolase activity (1.82 ± 0.80 µg/mL) were obtained for protein hydrolysates which were prepared with 5% (w/v) of neutrase at a molecular weight < 0.65 kDa. Meanwhile, Prakot et al.^19^. reported the highest level of TYR inhibition from peptides derived from the protein hydrolysates of the spotted babylon snail, for which the molecular weight was lower than 3,000 Da. Deng et al.^28^. reported similar findings, noting that greater TYR inhibition was exhibited by the smaller molecular weight fractions of the protein hydrolysates from Chinese quince seeds following UF. In line with this trend, the 0–3 kDa fraction was chosen to undergo additional purification. Pongkai et al.^18^. confirmed the benefits of using the UF membrane to enhance the activity of a peptide fraction in terms of TYR inhibition, since the inhibitory activity of a peptide is closely related to its molecular weight. Accordingly, the protein hydrolysates prepared using 5% (w/v) neutrase which has a molecular weight < 0.65 kDa were shown to offer strong TYR inhibition, since they were able to exert their inhibitory influence at low concentrations, thus exhibiting strong dependence upon molecular weight. The differences observed might be attributable to the susceptible bonds in the amino acid sequences, which might have been dependent on the mechanism for catalysis of the immobilized enzyme, the breaking point preference in the protein sequence, and protein isolates composition. The fraction with molecular weight < 0.65 kDa which offered strong mono-phenolase and di-phenolase inhibition was selected to be purified further using size exclusion chromatography **(**SEC) and reversed-phase high-performance liquid chromatography (RP-HPLC).

**Table 2:** TYR inhibition (IC_50_) results of BPPH ultrafiltered fractions.

Molecular weight (kDa)	L-Tyrosine (mono-phenolase activity) IC_50_ (µg/mL)	L-DOPA (di-phenolase activity) IC_50_ (µg/mL)
> 10	60.15 ± 1.66^c^	66.83 ± 5.56^c^
10–5	12.60 ± 1.65^b^	12.58 ± 1.07^b^
5–3	3.52 ± 1.22^a^	5.33 ± 1.90^a^
3-0.65	2.34 ± 1.31^a^	4.01 ± 2.66^a^
< 0.65	1.08 ± 0.50^a^	1.82 ± 0.80^a^

All values are presented in the form of TYR inhibition mean (IC_50_ value) ± SE. Deviation, with the tests performed in triplicate. When a letter in superscript is presented in the same column, this is showing significant differences (*p* > 0.05).

### TIP purification through SEC and RP-HPLC

**Fig. 1:**
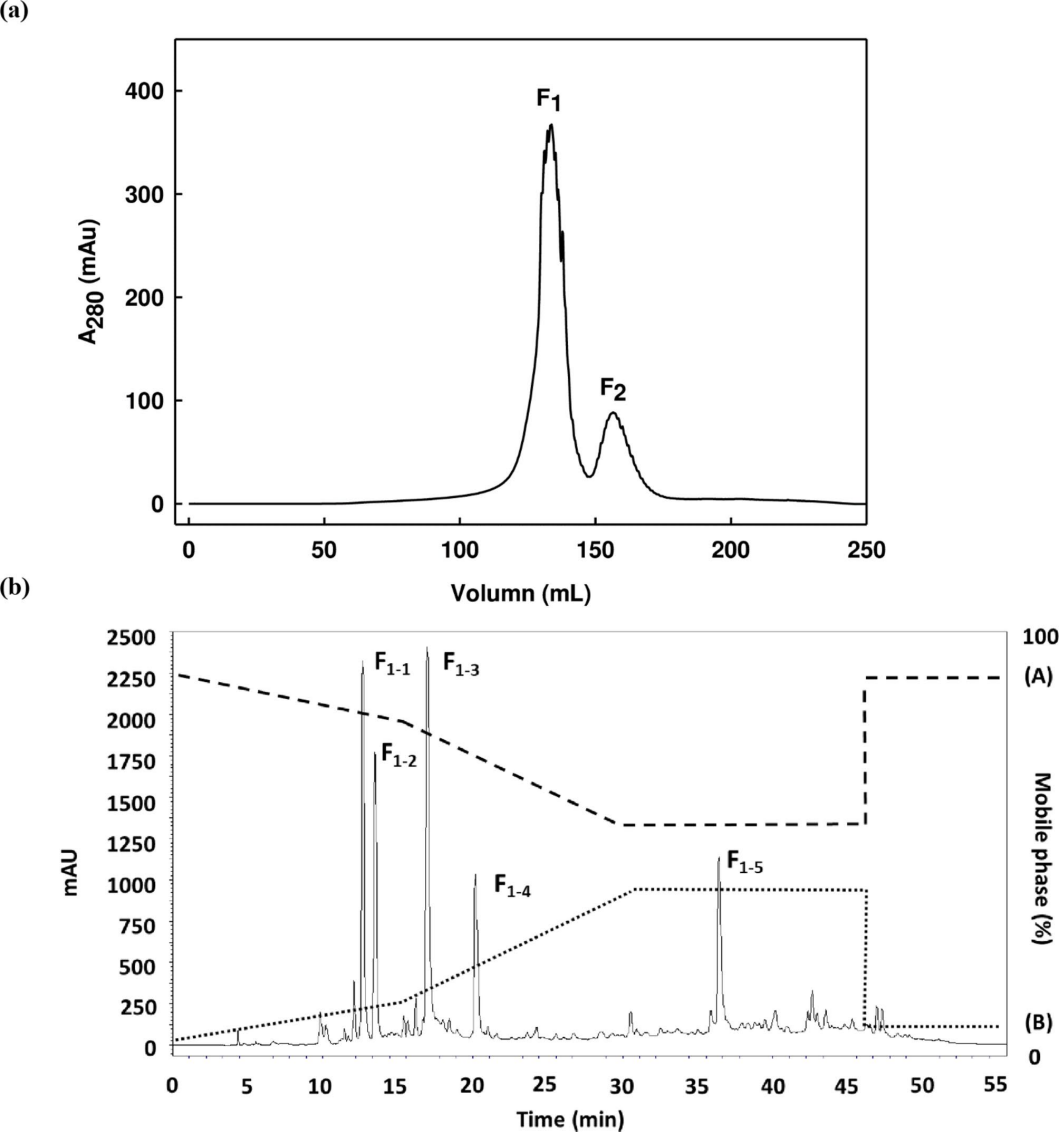
(**a**) SEC of the F_1_ and F_2_ fractions from MW < 0.65 kDa neutrase hydrolysate. (**b**) RP-HPLC profile of the active fraction (F_1_) obtained from BPPH.

Figure 1a serves to explain that higher levels of TYR inhibitory activity may result from the fraction with molecular weight < 0.65 kDa at a half-maximal inhibitory concentration (IC_50_) because of that low molecular weight. Further fractionation of the MW < 0.65 kDa ultrafiltrated fraction can be carried out by SEC using a Superdex 30 Increase column with a separation range of 100-7,000 Da. It is then possible to monitor the various peptide bonds, proteins, peptides, or amino acids with aromatic rings through the use of A_280_, which revealed two individual peaks (F_1_ and F_2_), confirming that aromatic peptide or amino acid side chains were present. F_1_ presented TYR inhibition for mono-phenolase with an IC_50_ value of 46.10 ± 1.25 µg/mL, and for di-phenolase with an IC_50_ value of 37.39 ± 1.26 µg/mL. However, it was not possible to calculate such values for F_2_. On the basis of these outcomes, the F_1_ fraction was chosen for subsequent analysis. This lyophilized active F_1_ fraction underwent additional separation using RP-HPLC to create the five sub-fractions indicated as F_1–1_, F_1–2_, F_1–3_, F_1–4_ and F_1–5_ (Fig. 1b). The inhibition of TYR varied among the various sub-fractions, with sub-fraction F_1–2_ exhibiting the highest levels of TYR activity in both mono-phenolase (IC_50_ = 17.28 ± 0.02 µg/mL) and di-phenolase (IC_50_ = 25.69 ± 0.30 µg/mL). Furthermore, the F_1–1_ and F_1–5_ sub-fractions featured very low protein concentration levels so it was not possible to calculate an IC_50_ value for the TYR activity (S1 Table). In the work of Kubglomsong et al.^29^. papain hydrolyzed rice bran albumin hydrolysates were reported to offer greater TYR inhibition, and were then separated into 11 fractions via RP-HPLC. The greatest level of TYR inhibition was demonstrated by Fraction 1 (IC_50_ = 1.31 mg/mL), which comprised 13 peptides with molecular weights in the range from 1.3 kDa to 4.8 KDa. One particular peptide displayed the amino acid sequence SSEYYGGEGSSSEQGYYGEG, and was noted for its residues and other features associated with TYR inhibition and chelating copper ions. It has been confirmed by a number of studies that TYR inhibition is linked to peptides’ amino acid composition and molecular weight distribution. Accordingly, it was necessary to investigate the amino acid sequence of the F_1–2_ fraction, which showed significant TYR activity, via liquid chromatography-quadrupole time-of-flight-tandem mass spectrometry (LC-Q-TOF-MS/MS).

### Identification and sequencing of isolated peptides and bioinformatic predictions of peptide properties

**Fig. 2:**
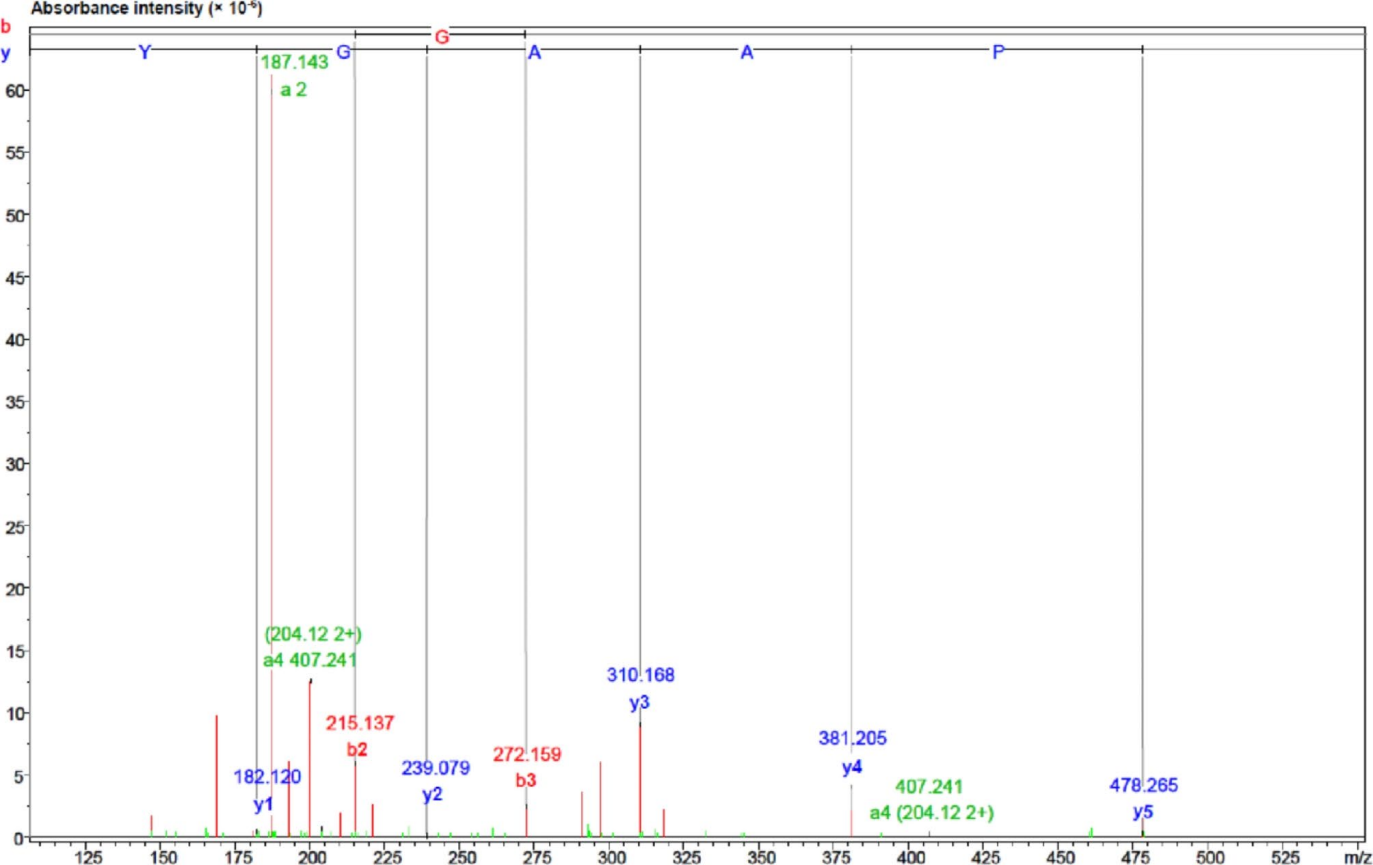
Mass fragmentation spectrum and analysis of the F_1–2_ fraction (VY-9) amino acid sequence via LC-Q-TOF-MS/MS.

The selected F_1–2_ fraction underwent LC-Q-TOF-MS/MS and *de novo* amino acid sequencing in order to evaluate its various characteristics, which could then be reassessed via the NCBI database in order to establish the identity in the *Mimosa* genus, as shown in Fig. 2. The VY-9 peptide, discovered through *de novo* sequencing, shows partial similarity to apyrase proteins from *Mimosa pudica*, suggesting a possible functional relationship while indicating novelty within the *Mimosa* genus. The F_1–2_ peptide (VDGYPAAGY; Val-Asp-Gly-Tyr-Pro-Ala- Ala-Gly-Tyr; named VY-9) matches to the peptide of nine amino acids with the molecular weight of 911.95 Da. Protein BLAST allowed the location to be established in the homologous region. Accordingly, the VY-9 peptide showed full similarity to the apyrase protein which can be obtained from *Mimosa pudica* (S2 Table). Apyrases are enzymes that serve to eliminate other enzymes which would extract the terminal phosphate from NTPs and NDPs, while not doing so in the case of nucleotide monophosphates. Their role in plant and animal physiology is highly varied^30^. The preparation of functional peptides requires a thorough comprehension of peptide properties and stability. One key property is the solubility of a peptide in water, since this will affect drug release, bioavailability, and absorption^31^. Water solubility is thus a critical property when considering the use of a particular peptide in the medical, cosmetic, or nutritional fields. The VY-9 peptide has poor water solubility according to the Innovagen server (Table 3), yet our own findings indicated that at concentration below 5.0 mg/mL the water solubility was not as poor as might be anticipated. However, as the concentration increased, the solubility did tend to become worse. Meanwhile, peptide or protein toxicity can be estimated in silico by ToxinPred, which is a very useful tool in the area of drug discovery^32^. In this case, ToxinPred was used to assess the toxicity of the peptide VY-9, with the outcome suggesting a lack of toxicity, and thus acceptability for applications in the food, medical, or cosmetic sectors. The findings can be seen in Table 3.

**Table 3:** The property profiles of the peptide VY-9.

List of properties	Peptide properties
Water solubility^a^	Poor
Hydrophobicity (%)^b^	44.44%
Toxicity (SVM score)^c^	Non-Toxic (− 0.42)

^a^Data obtained from the Innovagen server concerning peptide solubility.

^b^Calculations were performed on the peptide property calculator (www.peptide2.com).

^c^Outcomes from peptide toxicity analysis was performed using the ToxinPred server.

The peptide VY-9 comprises 44.44% hydrophobic amino acid residues. It is normally the case that low molecular weight protein hydrolysates exhibit greater hydrophobicity as well as superior dispersion which leads to stronger overall bioactivity. Where hydrophobic amino acids appear at each end of the peptide chain, this increases the interactivity with the copper active sites of TYR. Furthermore, it was reported by Wang et al.^33^. that a low-molecular-weight (700-1,700 Da) gelatin hydrolysate derived from the sea cucumber wall offered 55.7% hydrophobic amino acids. While the exterior environment of TYR is hydrophilic, the inner core forms a hydrophobic cavity such that those amino acids which contain a hydrophobic side chain will tend to bind more readily to the hydrophobic pocket close to the active site of TYR. Aromatic amino acids have benzene rings which can be buried at the active site as they bind to TYR, and since they are hydrophobic, this stabilizes the binding, increasing the inhibitory effects against TYR. According to Ge et al.^34^. three new peptides offering notable inhibitory activity against TYR include ILFTLL (IC_50_ = 9.25 mg/mL), TIPPPT (IC_50_ = 7.59 mg/mL), and IIPFIF (IC_50_ = 6.16 mg/mL). However, the peptide IIPFIF stands out through its aromatic amino acid located at the *C*-terminal enabling entry to the active pocket from the *C*-terminal, whereas in contrast there is no aromatic amino acid at either end of the peptide chain in the case of TIPPPT or ILFTLL. Instead, the IIPFIF peptide comprises solely hydrophobic amino acids and its larger portion enters the active pocket, whereas TIPPPT and ILFTLL only have one or two hydrophilic amino acids and the smaller portion enters the active pocket.

### Validation of synthetic peptide VY-9 activity

In order to check our own hypothesis concerning the ability of the peptide VY-9 to inhibit TYR, we created a synthetic peptide with a matching sequence to test the inhibition in the case of both mono-phenolase and di-phenolase forms. Our synthetic version of the peptide VY-9 demonstrated good TYR inhibition, for which the IC_50_ values of 0.55 ± 0.03, and 2.54 ± 0.06 µM were recorded in the case of the respective mono-phenolase and di-phenolase activities. The results indicated that the peptide VY-9 was more effective in the inhibition of mono-phenolase than di-phenolase, possibly because the inhibitory concentration threshold for the di-phenolase reaction could exceed that of the mono-phenolase reaction. In earlier research the protein from rice bran underwent enzymatic digestion with the simultaneous use of chymotrypsin and trypsin to create peptides. From the rice bran protein hydrolysates, six bioactive peptides were isolated and determined to exhibit TYR inhibitory activity. These peptides were classified as CT-1-6, and the amino acid sequence analysis determined that three of these peptides possessed tyrosine residue at the *C*-terminus side of the peptides (CT-1: HGGEGGRPY, CT-2: LQPSHY, and CT-3: HPTSEVY). These three peptides produced notable TYR inhibition in the mono-phenolase tyrosine substrate reaction, but importantly did not do so in the di-phenolase L-DOPA substrate reaction. Moreover, melanogenesis in melanoma cells was shown to be inhibited by the peptide CT-2 (> 50% at 500 µM) while no cytotoxic effects were recorded^35^. On the basis of these data, it could be argued that protein hydrolysates from rice bran might serve as an excellent source of TYR inhibitory bioactive peptides. The inhibitory activity was shown to be lower for the di-phenolase when compared to the mono-phenolase in a majority of the substances examined^36^. Meanwhile, Li et al.^37^. reported significant differences in the measured IC_50_ values between mono-phenolase and di-phenolase inhibitory activity towards mushroom TYR. Where such differences occur between the mono-phenolase and di-phenolase activity for a particular compound, it can most commonly be attributed to differences in the catalytic cycle mechanisms of monophenol (L-tyrosine) and diphenol (L-DOPA) oxidation by the mushroom TYR. It is not, however, possible to rule out the significant inhibition of mono-phenolase steady-state activity with no extension of the lag time. For TYR activity in the context of L-tyrosine as a monophenol substrate it is necessary to implement a lag-phase in order to achieve the transformation to diphenol, which acts as a substrate to di-phenolase.

VY-9 has *C*-terminal tyrosine residue which supports the TYR inhibition, possibly as a consequence of the aromatic ring structures in the amino acid Tyr residues. Ge et al.^34^. noting that hydrophobic interactions with hydrophobic side chains located at the active sites of TYR could be increased by inhibitors which have aromatic ring structures. Furthermore, a number of researchers have suggested that the tyrosine residue makes an important contribution to TYR inhibition. For instance, Schurink et al.^38^. performed the screening of seven different peptides offering TYR inhibition from protein-based peptide libraries via SPOT synthesis, with the results indicating that peptides which contained tyrosine were able to activate TYR since it was possible for them to undergo conversion by TYR. In addition, Ochiai et al.^35^. examined TH10 (MRSRERSSWY), synthesizing it following a search of the rice DNA database. The peptide TH10 was found to have an IC_50_ value of 102 µM. This peptide can be considered homologous to peptide P4 (YRSRKYSSWY) from an earlier study which achieved an IC_50_ value of 123 µM where tyrosine was a substrate of TYR. These peptides both have sequences containing tyrosine on the *C*-terminal, while peptide P4 has an additional two tyrosine residues, located in the middle of the peptide and at the *N*-terminal. Simulations were performed to assess the sequence-shuffled variants of peptide P4 and the molecular docking of variants of peptide TH10, revealing that the peptide P4 inhibitory activity was not linked to the presence of tyrosine residues at the center of the peptide sequence and in the *N*-terminal position, although the tyrosine residue at the *C*-terminal in both of the peptides is important in TYR inhibition because tyrosine residue on both peptides plays very important role in the inhibition activity of TYR by acting as a substrate analogue at the TYR active site and coordinating with the copper ions of the TYR enzyme. Most peptides offering TYR inhibition have Tyr residue at the *C*-terminal.

### Inhibition type and constant determination for the VY-9 peptide

**Fig. 3:**
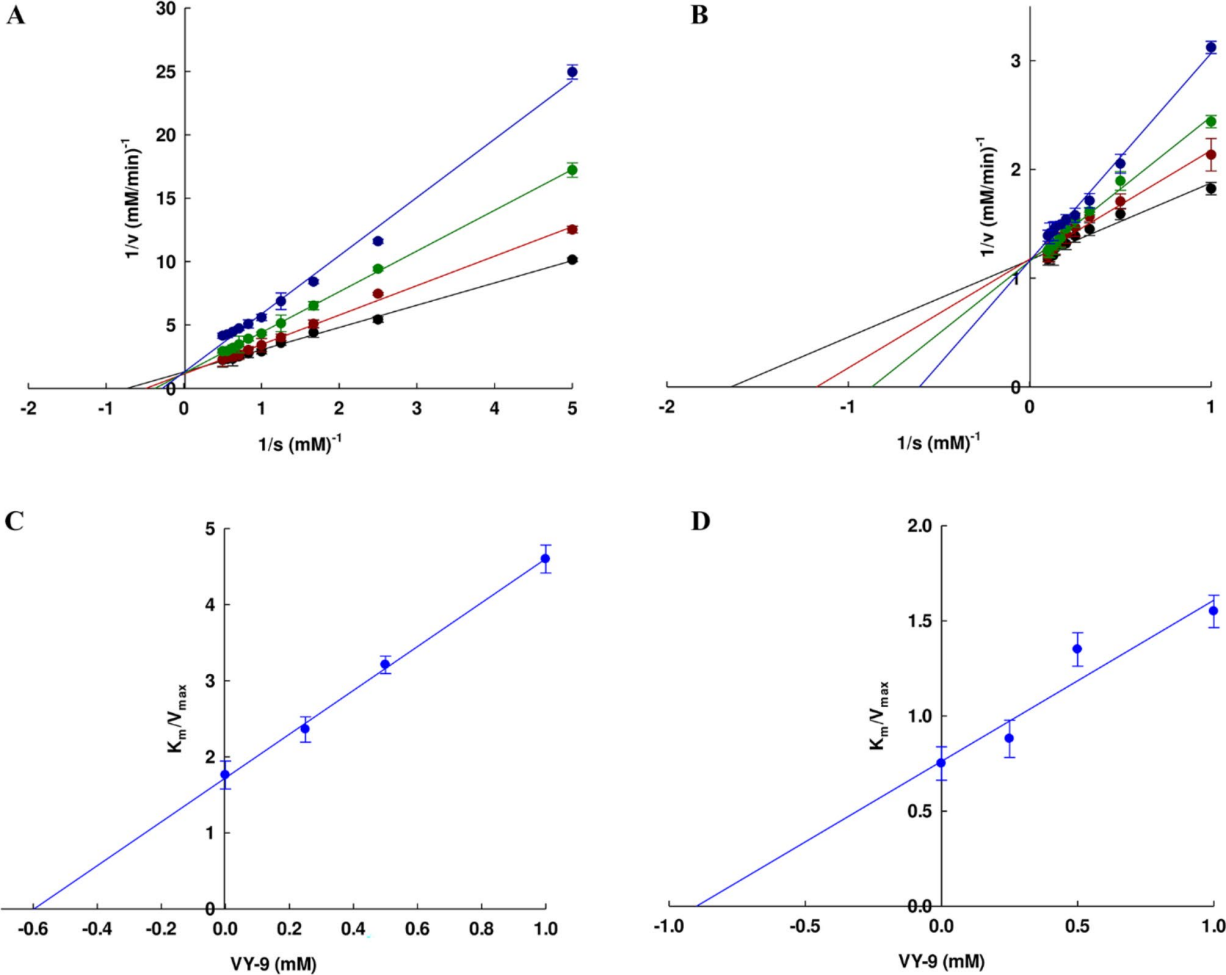
The Lineweaver-Burk plot of the peptide VY-9 when inhibiting (**a**) mono-phenolase activity and (**b**) di-phenolase activity. Reactions were carried out at varying inhibitor concentrations: No inhibitor (filled black circle), 0.25 mM (filled red circles), 0.5 mM (filled green circle), and 1.0 mM of VY-9 (filled blue circle). Lineweaver-Burk secondary plots revealed the determination of the inhibitor constant (Ki) in inhibiting (**c**) mono-phenolase activity and (**d**) di-phenolase activity by the peptide VY-9.

In order to understand the VY-9 peptide mechanism for TYR inhibition, the inhibition mode was investigated through the analysis of Lineweaver-Burk plots. The plots can be seen in Fig. 3a-d, where four lines of differing gradients meet at the same intersection point for the vertical coordinate at varying concentrations of the VY-9 peptide. The results show that the Km values rose as the concentration of the inhibitor rose, whereas the Vmax values remained unchanged suggesting that the peptide VY-9 acted as a competitive inhibitor in the case of tyrosine mono-phenolase and also for di-phenolase. In this way, the peptide VY-9 was capable only of binding to free enzymes, but could not form bonds with enzyme-substrate complexes. The inhibition constant Ki had a value of 0.6 ± 0.09 mM for tyrosine mono-phenolase and 0.82 ± 0.07 mM for di-phenolase. Feng et al.^39^. conducted similar investigations, determining that the inhibitory activity of the peptide FPY, a competitive inhibitor obtained from de-fatted walnut, had a Ki value of 22.04 ± 0.09 mM for mono-phenolase and 4.82 ± 0.07 mM for di-phenolase. Meanwhile, the Phage Display Library revealed the peptide IQSPHFF to act as a competitive inhibitor for di-phenolase, with the Ki value reported to be 0.765 mM^40^. In other studies, Yap and Gan reported that GYSLGNWVCAAK, obtained from egg white protein, could competitively inhibit TYR^13^.

### Molecular docking

**Fig. 4:**
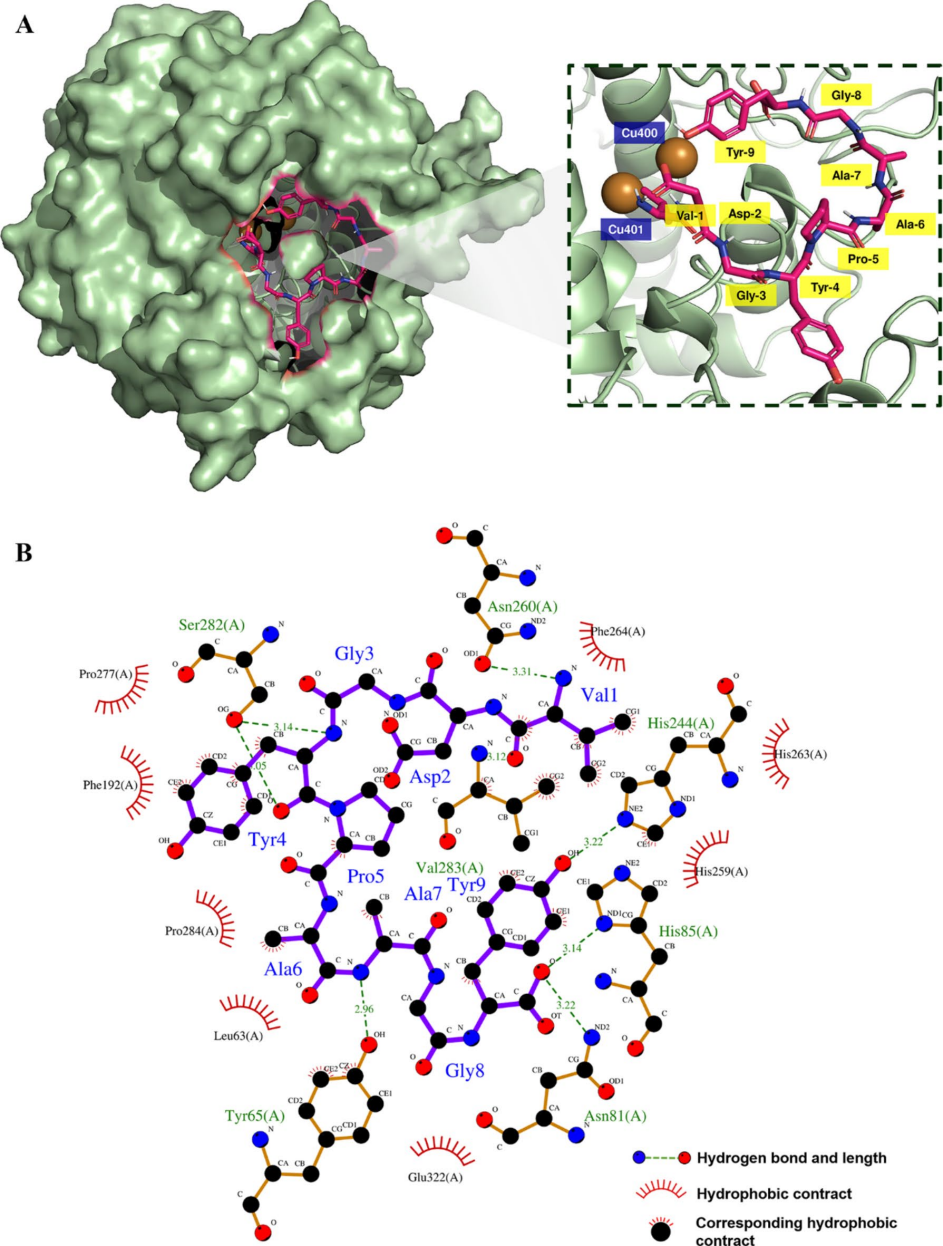
The molecular docking model for the peptide VY-9 and TYR. (**a**) The 3D map of the peptide VY-9 docking at the TYR activity center. (**b**) The 2D projection of the peptide VY-9 and TYR docking model. This image was produced using LigPlot.

Studies of molecular docking via computer simulation have recently become increasingly widely used. The technique allows the mechanisms by which inhibitors and enzymes interact to be investigated^41^. In this study, it was necessary to better understand the peptide inhibition mechanism in the context of TYR, so molecular docking was employed to examine the peptide VY-9 interacting with TYR. The results can be observed in Fig. 4a,b. When comparing the findings, it was evident that the VY-9 formed effective bonds to the peripheral residues of TYR via hydrogen bonds, hydrophobic interactions, van der Waals forces, and π-σ bonds. In another study, Ismaya examined the crystal structure of the mushroom TYR (abTYR) obtained from *Agaricus bisporus*, revealing that abTYR has a crystal structure which is an H2L2 tetramer comprising two H subunits and two L subunits. There are 150 subunits in the L subunit which is the result of an unrelated gene which plays an undetermined role. In contrast, the H subunit may derive from the TYR ppo3 gene since it comprises 391 amino acid residues which perfectly match the residues 2-392 of ppo3. The H subunit has an active binding site for abTYR which has two copper atoms (Cu400 and Cu401) along with six histidines (His). CuA will bind to the His61, His85, and His94 ligands, while CuB will bind to the His259, His263, and His296 ligands^42^. As a consequence of these histidine residues undergoing ligation with copper ions, the rotational freedom is restricted. The integrity of the binding site, Phe90, located between His94, His259, and His296, and Phe292, located between His61, His263, and His296, is maintained through constraining the histidine side chain conformation. Accordingly, it is clear that His61, His85, His94, His259, His263, His296, Phe90, and Phe292 are vital amino acid residues which affect TYR activity.

In order to predict the potential protein–peptide binding interactions, AutoDock Vina molecular docking analysis was first conducted. Then to predict the protein–peptide binding affinity, the LigPlot^+^ 2.0 server was employed. Further study of molecular docking was then performed to determine how the peptide VY-9 interacts with the TYR activity pocket. The interaction model deemed to be most probably for the peptide VY-9 binding with TYR displayed a binding energy of − 8.3 kcal mol^− 1^. In its central activity region, the peptide VY-9 could use hydrogen bonds at the residuals Tyr65, Asn81, His85, His244, Asn260, Ser282, and Val283 to interact with TYR, along with hydrophobic interactions with Phe264, His263, H259, Pro277, Phe192, Pro284, Leu 63, and Glu322. Direct chelation with the copper ions is observed for the residue His85, and when the tyrosine residue oxygen atom on the peptide VY-9 bonds with the His85 hydrogen atom, this will influence enzyme activity, placing the tyrosine residue near to the copper ions, indicating that the peptide VY-9 binds to TYR as a substrate analogue, preventing melanin production.

The VY-9 peptide contains tyrosine which has a benzene ring that performs powerful hydrophobic interactions with Pro277 and Phe192, potentially blocking the TYR hydrophobic pocket, and lowering its hydrophobicity. In addition, the docking simulation reveals that the tyrosine residues of CRY and RCY interact with the Trp227 and Met257 side chains via hydrophobic interactions which serve to cover the TYR active pocket. While the exterior environment of TYR is hydrophilic, the inner core forms a hydrophobic cavity such that those amino acids which contain a hydrophobic side chain will tend to bind more readily to the hydrophobic pocket close to the active site of TYR. Furthermore, aromatic amino acids have benzene rings which can be buried at the active site as they bind to TYR, and since they are hydrophobic, this stabilizes the binding, increasing the inhibitory effects against TYR^43^. When comparing the binding of arbutin and the VY-9 peptide with TYR, the VY-9 peptide exhibited the lowest binding energy at -8.3 kcal/mol, while arbutin showed a binding energy of -6.5 kcal/mol (S2 Figure, and S3 Table). Furthermore, the VY-9 peptide engages with seven binding residues: Tyr65, Asn81, His85, His244, Asn260, Ser282, and Val283. In contrast, Arbutin interacts with only four residues: Gln144, Glu173, Asn174, and His178. This result suggests that the VY-9 peptide binds to TYR more strongly than arbutin. In previous research there was a proposal for the inhibition mechanism for “tyrosine-type” TYR inhibitory peptides, including those similar to TH10. Furthermore, docking simulation has indicated that these peptides serve as substrate analogues which block access to the TYR active site for L-tyrosine. A simulation was also carried out using the mushroom TYR structure to determine the peptide CT-2 binding mode. These investigations revealed that the *C*-terminal tyrosine residue of CT-2 is bound to TYR active site with its copper through a mechanism similar to that of TH10, which would indicate that there is a strong affinity for CT-2 to the TYR active site, while CT-2 also serves as a substrate analogue. It is not, however, clear exactly why CT-2 proved more effective than the other isolated peptides in terms of inhibitory activity. Previous reports have suggested that while the tyrosine residue at the *C*-terminal plays a major inhibitory role in “tyrosine-type” TYR inhibitory peptides, there may also be a significant contribution made by the nearby amino acid residues^36^.

### The influence of the peptide VY-9 on B16F10 melanoma cells

**Fig. 5:**
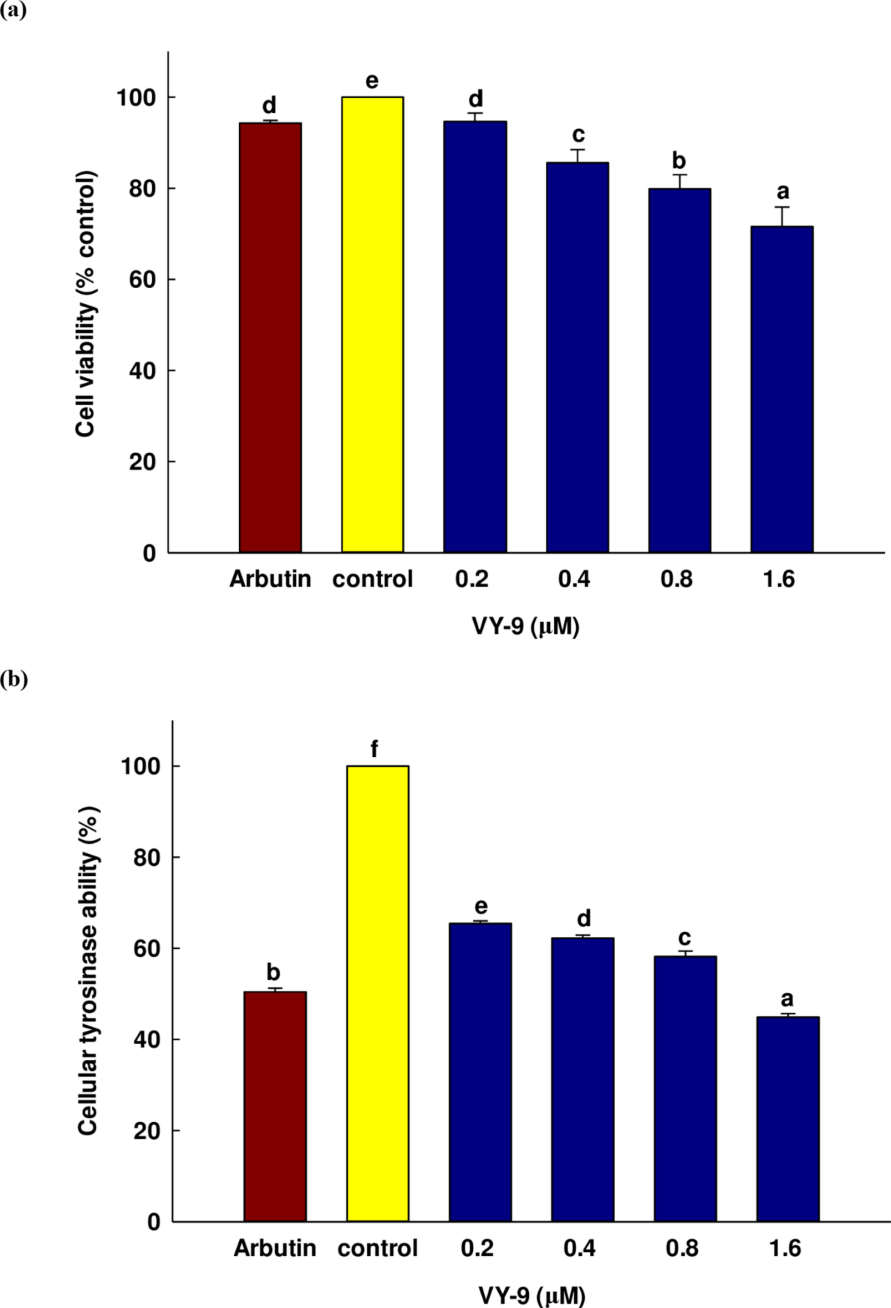

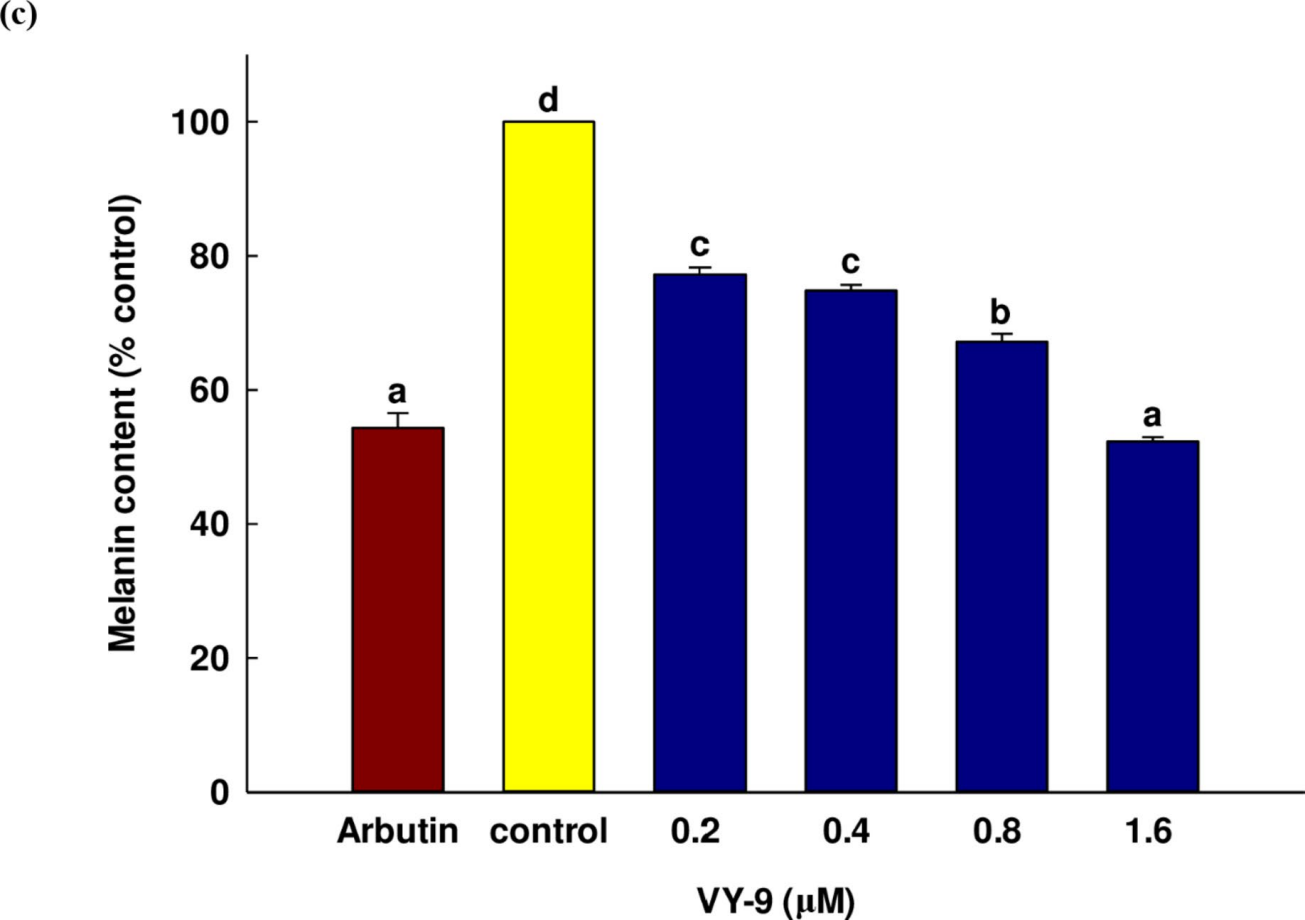
The influence of the peptide VY-9 on B16F10 melanoma cell cytotoxicity (**a**), tyrosinase activity (**b**), and melanin content (**c**). The positive control used was arbutin (0.2 mM). The findings are presented in the form of percentage of the control, while the values represent the mean ± SD from three independent replications. For Duncan’s test, the different letters are used to denote a significant statistical difference between groups when *p* < 0.05.

In producing cosmetic products and functional foods, safety is of the highest priority during the formulation process. For instance, while hydroquinone is widely used in the treatment of hyperpigmentation and serves as a common skin-whitening treatment, it is known to induce a number of undesirable side effects such as contact dermatitis, irritation of the skin, and exogenous ochronosis in individuals of a darker skin tone. For these reasons, hydroquinone cannot be used as a cosmetic component in the European Union, and is strictly supervised by the Food and Drug Administration in the USA^44^. Accordingly, this study commenced by assessing the cell viability of the peptide VY-9 in order to estimate the safety. The cells investigated in vitro included B16F10 melanoma cells from mice. Many studies focusing on pigmentation make use of the B16F10 melanoma cell line, since TYRs in humans and mice tend to be homologous with 86% identity at the protein level and 84% at the cDNA level. Moreover, these cell lines regulate the mechanisms of melanin synthesis in a similar manner to that of human melanocytes. Also, the cells are able to become attached to the culture flask and will create homogenous cell populations. It can then be feasible to observe the cellular differentiation from the perspectives of both morphology and melanogenesis^45,46^. The cytotoxicity of the various concentrations of the peptide VY-9 was evaluated using the MTT colorimetric assay, and comparisons were drawn with arbutin (0.2 mM). The influence of the peptide VY-9 peptide with arbutin was examined in the context of B16F10 cell viability, with the outcome shown in Fig. 5a. As the peptide concentration rose, there was a decline in the survival rate of the cells. However, inside the concentration range used for testing, from 0 to 1.6 µM, there were no clear signs of cytotoxicity (viability > 50%). The ISO 10993-5:2009 guidelines suggest that any treatment which leads to a reduction in cell viability of more than 30% can be classified as toxic^47^.

For additional investigation of the TYR activity which takes place in B16F10 melanoma cells after treatment by the peptide VY-9, different concentrations of the peptide VY-9 were used along with arbutin (0.2 mM). Figure 5b presents the outcomes. We are not aware of any previous studies examining mono-phenolase activity which make use of TYR derived from cell models. The lack of research in this area may be due to the need to initially accumulate L-DOPA *via* di-phenolase activity in order to stabilize TYR so that its catalytic capabilities can be fully activated. TYR can be found in three different oxidation forms, which in turn present different capabilities in phenol catalysis or the catalysis of catechol substrates. Meanwhile, mono-phenolase activity kinetics can be complex and are not east to observe^48^. As a consequence, we did not examine the mono-phenolase reaction in this work. As the concentration of the peptide VY-9 was increased, the TYR activity began a steady decline, suggesting that TYR inhibition was increasing. This rate of inhibition was measured at 44.88% using 1.6 µM of VY-9, exceeding the performance of arbutin which offered 50.45% inhibition at 0.2 mM. Although molecular docking predicted that VY-9 interacts with the tyrosinase catalytic site, further experimental validation is required to confirm the peptide’s ability to penetrate cell membranes and reach intracellular tyrosinase. Future studies will involve permeability assays, such as Franz diffusion or cellular uptake studies^49^, to verify the peptide’s intracellular delivery.

In skin, melanin content determines the level of whiteness. It is understood that melanin is produced when tyrosine is oxidized to form DOPA due to the action of TYR, whereupon DOPA undergoes further oxidization to DOPA-quinone, which in turn undergoes a number of reactions which create eumelanin. The TYR enzyme is therefore crucial in producing melanin. Figure 5c shows the steady decline in melanin content as peptide VY-9 increased in concentration, revealing a relationship strongly dependent on concentration levels and confirming the ability of VY-9 to restrict melanin production. The inhibition level was shown to reach a 54.34% reduction in melanin content when using arbutin (0.2 mM) to treat the cells, while the peptide VY-9 achieved a 52.31% reduction at 1.6 µM. While the arbutin rate was better, melanin synthesis is influenced by a number of factors in addition to TYR, and it would appear that arbutin is able to regulate the transcription of TYR pathway genes, bleaching the melanin which is produced^50^.

Abu Ubeid et al.^51^. conducted the screening of an internal library to identify the active oligopeptides capable of inhibiting TYR in mushrooms. Notably, the oligopeptides YRSRKYSSWY (IC_50_ = 40 µM) and RADSRADC (IC_50_ = 123 µM), showed greater activity than hydroquinone (IC_50_ = 680 µM). However, some peptides, such as KFEKKFEK (IC_50_ = 3.6 mM) and SFLLRN (IC_50_ = 8 mM) showed lower levels of activity. Meanwhile, human TYR was also inhibited by the peptides YRSRKYSSWY and RADSRADC to a greater extent than was the case with hydroquinone. When human melanocytes underwent treatment for seven days with YRSRKYSSWY and RADSRADC at 100 µM there was a reduction in melanin content by 43% for the former and 27% for the latter. In addition, a docking study based on a library of short sequence oligopeptides in the context of the mushroom TYR crystal structure allowed the identification of several oligopeptides which displayed favorable binding free energies and interacted directly with the catalytic enzyme pocket. In the work of Shen et al.^52^. the TYR inhibition of the peptide ECGYF was reported, with the peptide comprising a short sequence of the protein midasin (MDN1) which is obtained from Vigna seeds. The reported TYR inhibition capabilities of ECGYF (IC_50_ = 0.46 mM) exceeded those of arbutin and glutathione and the peptide (0.5-1 mM) was also able to lower the melanin levels in cultured A375 melanoma cells more effectively than arbutin or glutathione in the absence of cytotoxicity.

### Effect of the peptide VY-9 on gene and protein expression of the melanogenesis pathway in B16F10 cells

**Fig. 6:**
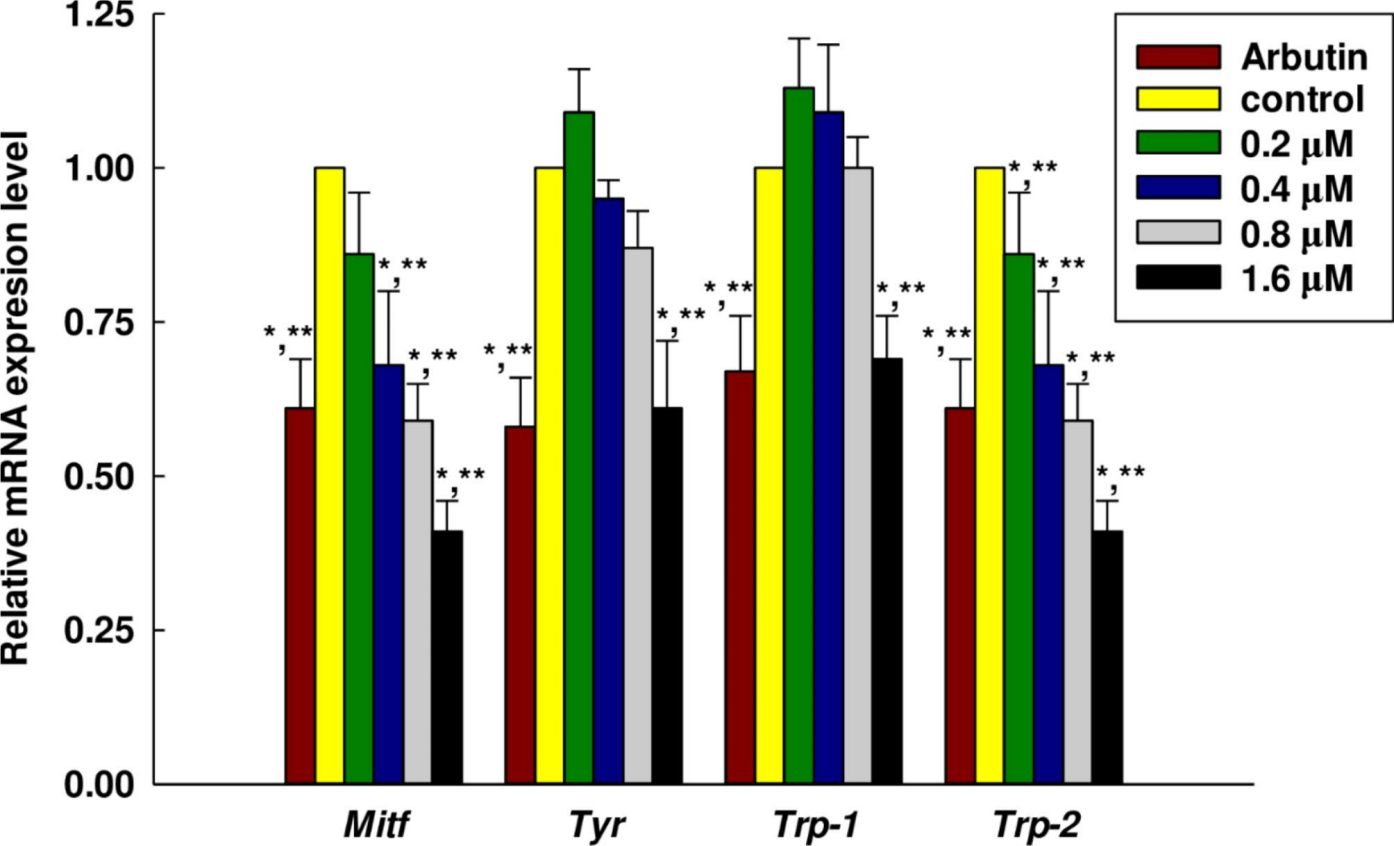
The mRNA expression levels of *Mitf*, *Tyr*, *Trp-1*, and *Trp-2* in B16F10 cells treated with different concentrations (0.2, 0.4, 0.8, and 1.6 µM) of the peptide VY-9. Results are presented as a percentage of the control, with values representing the mean ± SD of three independent replicates. All data are shown as mean ± SD. Post hoc analysis was conducted using Dunnett’s test. Significant differences compared to the control group are indicated as follows: *p < 0.05 and **p  <  0.01. Arbutin (0.2 mM) was used as the positive control.

**Fig. 7:**
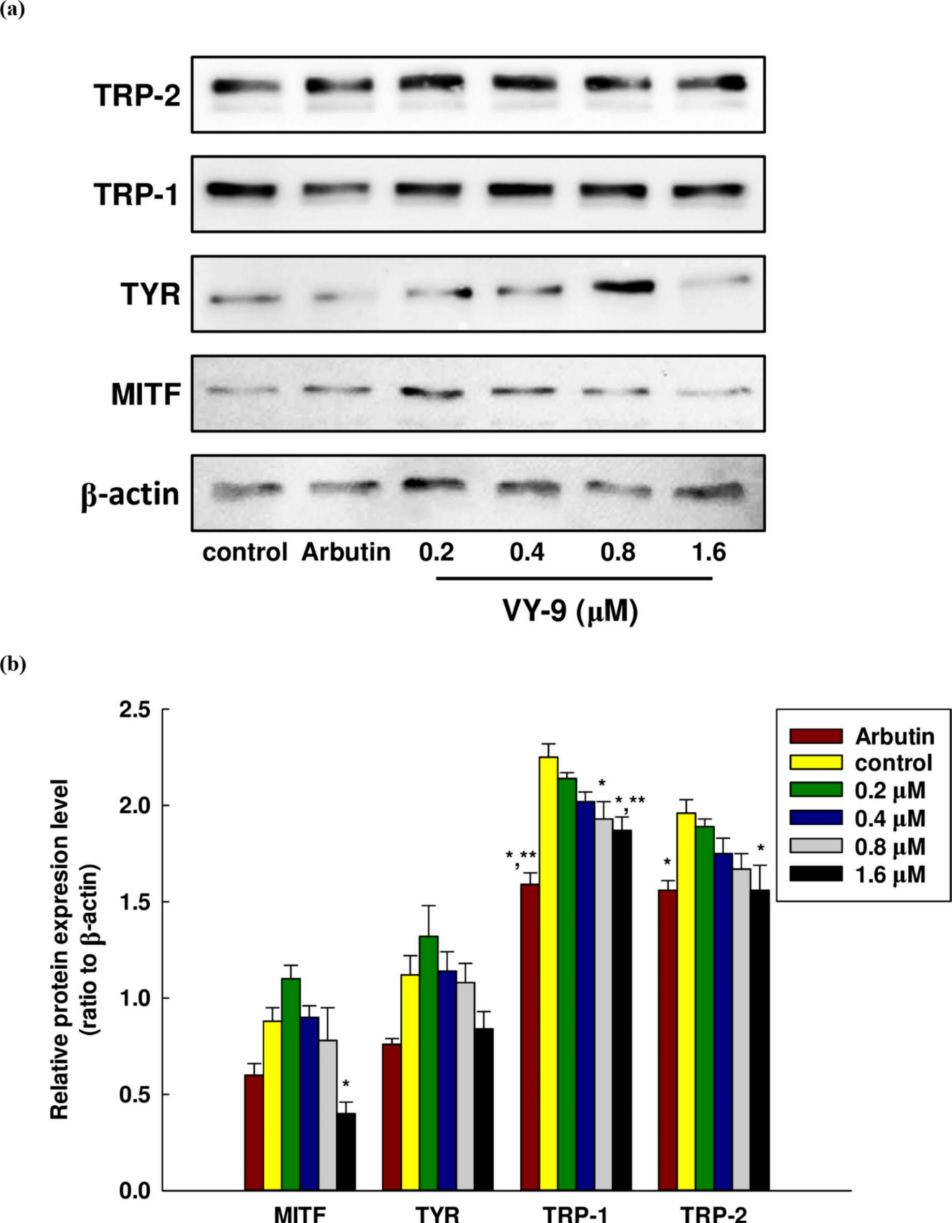
Western blot analysis was conducted for the comparison of melanin-related protein expressions in B16F10 cells in both the presence and absence of the peptide VY-9 for 48 h at concentrations of 0.2, 0.4, 0.8, and 1.6 µM. (**A**) Western blotting; and (**B**) Relative intensity of the protein band quantified by ImageJ software, with the value normalized to match the corresponding loading control. All data are shown as mean ± SD. Post hoc analysis was conducted using Dunnett’s test. Significant differences compared to the control group are indicated as follows: *p < 0.05 and **p < 0.01. Arbutin (0.2 mM) served as the positive control.

MITF (microphthalmia-associated transcription factor) was a basic helix-loop-helix leucine zipper transcription factor relative to line age specific pathway regulation of certain cell models including osteoclasts, melanocytes, and mast cells. Importantly, MITF was the transcription factor of TYR, TRP-1, and TRP-2 in the melanogenesis pathway. Furthermore, in melanin production, TYR was an oxidase serving as the rate-limiting enzyme which controlled the production of melanin, while TRP-1 was a melanocyte-specific gene associated with melanin synthesis. In addition to this role, TRP-1 also served to stabilize the TYR protein, governing its catalytic abilities^53^. The expression of TRP-1 and TRP-2 was altered by the MITF, and the influence of the peptide in inhibiting the expression of melanogenesis was examined using qRT-PCR. The qRT-PCR data presented in Fig. 6 revealed that once the B16F10 cells had undergone treatment with the peptide, at a concentration of 1.6 µM, peptide VY-9 significantly reduced the expression levels of all measured genes relative to the control. For *Mitf* and *Trp-2*, expression decreases were observed starting from the lowest concentration of 0.2 µM. In contrast, *Tyr* expression began to decrease at 0.4 µM, while *Trp-1* showed a reduction only at the highest concentration of 1.6 µM. Notably, at a peptide concentration of 1.6 µM, the expression of *Mitf* and *Trp-2* significantly decreased compared to arbutin, which served as a positive control. The protein expression results of the four target proteins measured by western blot were consistent with the gene expression results, as can be observed in Fig. 7a, b, and Fig. S1. At a concentration of peptide VY-9 at 1.6 µM, the expression of all proteins decreased compared to the control. However, compared to arbutin, only MITF showed a lower expression level. Starting from a concentration of 0.2 µM, the protein expression of TRP-1 and TRP-2 gradually decreased with increasing concentration. In contrast, the protein expression of MITF and TYR began to decrease at a concentration of 0.4 µM onwards.

During the course of our experiments, the highest concentration used was 1.6 µM, which reduced cell toxicity and effectively restricted the production of melanin and gene expression. Treatment with 1.6 µM resulted in a reduction in the gene and protein expression levels of MITF, TYR, TRP-1, and TRP-2, aligning with the findings for melanin content at that same concentration, as shown in Fig. 5c. Notably, at the lowest peptide concentration (0.2 µM), TRP-2 mRNA and protein levels decreased slightly compared to the control. The inhibitory effect became stronger as the concentration increased, as illustrated in Figs. 6 and 7. That is, TRP-2 activity could be influenced by additional physiological factors as well as the peptide itself. The main role of TRP-2 lies in converting L-dopachrome to DHICA in the melanogenesis pathway. The peptide examined in this study was able to inhibit the upstream transcription factor of TRP-2, but when the concentration of the peptide was increased, there was a fall in TRP-2 expression. This might arise as a consequence of MITF inhibition, and it could significantly change both TYR and TRP-1. We believed this was because the peptide could inhibit TYR and TRP-1 transcription, while a further possibility was that declining MITF expression might prevent TRP-2 from expressing normally. However, it was not possible to establish the precise reason for TRP-2 alterations which might allow the VY-9 peptide mechanism to be determined. While the peptide was capable of promoting TRP-2 expression, TYR was still significant as the rate-determining step. Furthermore, melanin synthesis must also occur *via* TRP-1, which was inhibited. We therefore take the view that this peptide would be capable of inhibiting the production of melanin. The peptide has also shown strong inhibitory capability based upon the dosage used in the inhibition of mushroom TYR, so enzyme activity inhibition may potentially be another outcome. These findings suggest that the peptide VY-9 may support skin whitening through the inhibition of melanogenesis via the obstruction of the mRNA and protein expression pathway.

### Effects of the peptide VY-9 on zebrafish embryos

**Fig. 8:**
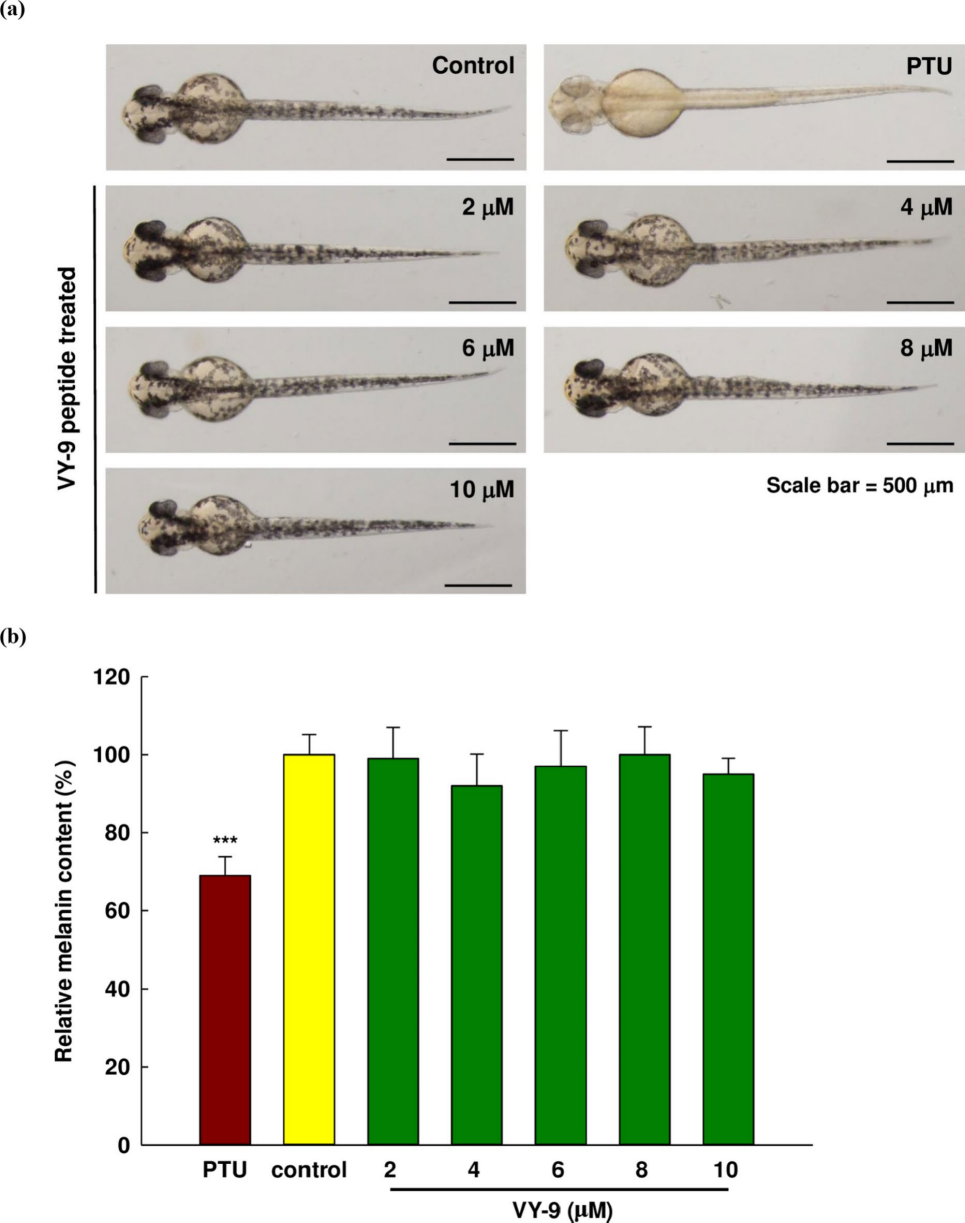
Influence of the peptide VY-9 upon melanin pigments in zebrafish embryos at 48 h after fertilization. (**a**) Representative zebrafish embryos under treatment with the peptide VY-9. Scale bar = 500 μm. (**b**) Quantitative analysis zebrafish embryo melanin content after treatment using the peptide VY-9. The findings are presented as percentage of the control. Data from three independent replications are shown in the form of mean ± SD.

This study has a number of different models to evaluate melanogenesis, and there are pros and cons to each of these models. For instance, the B16F10 model is inexpensive and allows the rapid assessment of de-pigmentation capabilities in an assay which permits high throughput, yet there are differences in the post-translational modification of TYRs when compared to the situation in humans, while the lack of metabolic activity which would typically be enriched in the epidermal layer can lead to inaccuracies in assessing the de-pigmentation efficacy. In such scenarios it would be necessary to carry out further investigation in human melanocytes and in models which more accurately represent the human metabolism. The zebrafish model was recently implemented for in vivo studies of melanogenesis^54,55^. It offers several advantages such as high levels of drug penetration, ease of maintenance, and does not require the application of stringent ethical rules which limit cosmetics testing on animals. The zebrafish provides an accepted vertebrate model for testing in the fields of toxicology and pharmacology. Zebrafish can be externally fertilized, are highly fecund, and the embryos develop quickly while offering transparency. The genetics and physiology of zebrafish are relatively similar to those of humans and other mammals^56^. Melanin can be synthesized in zebrafish, and it is possible to observe the pigment visually at around 28 h after fertilization. Accordingly, zebrafish embryos can readily serve as a model when testing melanin inhibition in various compounds^57–59^. In this research, we used zebrafish embryos to investigate the peptide VY-9 for melanin inhibition and also safety. Empirical determination guided the choice of concentrations examined in this study (2–10 µM). In zebrafish embryos, the peptide VY-9 displayed a “no observed effect concentration” (NOEC) of 10 µM. However, at 20 µM, the development of the embryo was significantly retarded. In this case, we found no significant difference in the rates for survival or malformation in the zebrafish embryo sample groups undergoing treatment when compared to the control group. These results suggest that the concentrations used for the in vitro analysis were safe when used with zebrafish embryos. Furthermore, embryo malformation was not observed when using these concentrations. Embryo samples following exposure can be seen in Fig. 8a. Additionally, the quantitative evaluation of melanin content showed no significant reduction in melanin levels at any concentration of the peptide VY-9 compared to the control. Although the relative melanin content at 4 µM was the lowest among all treatment groups, it did not achieve statistical significance compared to the control group, as depicted in Fig. 8b.

It was also shown that in embryos treated with PTU, the melanin content differed significantly (*p* < 0.01) to the control. These conflicting outcomes might be a consequence of the complexity of the organism involved. Since the zebrafish are able to absorb compounds comprising small molecules within the embryo medium through the skin, it may be possible for the peptide VY-9 to be exposed to biomolecules, potentially disrupting the inhibition of melanin synthesis^60,61^. This study has produced in vitro and in vivo findings which demonstrate the ability of the peptide VY-9 to inhibit melanin, although it may be more suitable for use in cosmetic products rather than nutraceuticals. That is, the inhibition of melanin synthesis might be more readily accomplished by the direct application of the peptide VY-9 to the skin, since this may offer greater efficacy than using the same peptide in the form of a supplement or drug. However, in a nutraceutical context, it is necessary to further examine the metabolism of the peptide VY-9 after entry to different organisms.

## Materials and methods

### Biomaterials

The bee pollen in this study came from *Apis mellifera* and was gathered in Chiang Mai, northern Thailand, from mimosa (*Mimosa pigra* L.) flowers in 2020 district ranging from 18°46’37.3”N latitude and 99°03’45.3”E longitude. The pollen was then placed in storage at 25℃ until required for analysis. The American Type Culture Collection provided the B16F10 murine melanoma cell line which was then cultured in Dulbecco’s Modified Eagle Medium (DMEM) (Gibco/Invitrogen, Carlsbad, CA) which contained 10% Fetal bovine serum (FBS) (Gibco/Invitrogen, Carlsbad, CA) and 100 U/mL of penicillin/100 µg/mL of streptomycin (Gibco/Invitrogen, Carlsbad, CA) at 37 °C in an incubator atmosphere of humidified 95% air/5% CO_2_. A recirculating tank system (AAB-074, Yakos65, Taiwan) was used to accommodate adult wild-type specimens of zebrafish at the National Nanotechnology Center (NANOTEC) National Science and Technology Development Agency, Thailand. Reverse osmosis water was supplied for the fish with the pH value maintained at around 6–8, with conductivity of 400–700 µS. In line with standard breeding protocols, the zebrafish were raised under a 14/10 h light/dark cycle at 28 ± 1 °C. Following natural pairwise mating, the embryos were gathered and placed in E3 medium (1 mM NaCl, 3.4 µM KCl, 6.6 µM CaCl_2_, and 6.6 µM MgSO_4_). Testing of normal fertilized eggs was then carried out using a stereomicroscope (SZX7, Olympus, Tokyo, Japan). The NSTDA Institutional Animal Care and Use Committee granted approval for the protocols used in the study (Approval No. 005-2562). We followed the Guiding Principles in the Care and Use of Animals of the American Physiology Society during zebrafish maintenance and experimentation.

### Chemicals

Arbutin, bovine serum albumin (BSA), L-3,4-dihydroxyphenylalanine (L-DOPA; catalogue number: D9628), dimethyl sulfoxide (DMSO), 3-(4,5-dimethyl-2-thiazolyl)-2,5-diphenyl-2 H-tetrazolium bromide (MTT), pancreatin from porcine pancreas (8 × USP specifications), kojic acid, (EC 1.14.18.1) (lyophilized powder, catalogue number: T3824), papain from papaya latex (≥ 10 U/mg), pepsin from porcine gastric mucosa (≥ 250 U/mg), and mushroom TYR (EC 1.14.18.1) (lyophilized powder, catalogue number: T3824), and L-tyrosine (catalogue number: T3754) were supplied by Sigma-Aldrich, Merck (St. Louis, MO, USA), while Brentag (Mülheim, Germany) provided protease from *Bacillus licheniformis* (EC 3.4.21.62; alcalase; ≥ 2.4 U/g), protease from *B. amyloliquefaciens* (EC.3.4.24.28; neutrase; ≥ 0.8 U/g), and protease from *Aspergillus oryzae* (EC 3.4.11.1; flavourzyme 500 MG). Thermo Fisher Scientific (San Jose, CA, USA) supplied formic acid, ethanol, acetonitrile (ACN), and trifluoroacetic acid (TFA), of chromatographic grade in all cases, while all chemicals listed were of analytical grade.

### BPPH preparation

To prepare BPPH, a mixture was created using 0.5 g of bee pollen and 10 mL of 20 mM phosphate buffer at pH 7.2. This mixture underwent overnight agitation at 4 °C with 150 mM NaCl. Hydrolysis was then carried out using differing quantities (1.0%, 2.5%, and 5.0% (w/v)) of alcalase, neutrase, flavourzyme, papain, or pepsin-pancreatin^62^. The sample used as the control contained no additive. All samples underwent activation in line with the process set out in Table 4 for the applicable enzyme conditions. After activation, the temperature increased rapidly to 80 °C to bring the enzyme activity to a halt. The hydrolysates obtained were then placed in a centrifuge at 15,900×*g* for 30 min at 4 °C, whereupon the supernatants were gathered and placed in storage at -20 °C prior to further experimentation.

**Table 4:** Conditions for the enzymatic hydrolysates from bee pollen.

Condition	Alcalase	Flavourzyme	Neutrase	Papain	Pepsin/pancreatin
% (w/v)	1, 2.5, 5	1, 2.5, 5	1, 2.5, 5	1, 2.5, 5	1, 2.5, 5
pH	8.0	7.0	7.0	7.0	2.5/7.5
Temperature (°C)	50	50	50	60	37
Time (h)	4	4	4	6	3/3

### Protein content determination

The Bradford procedure^63^ was employed to evaluate the protein hydrolysates which were obtained in the study in order to establish the protein concentration and to draw comparisons to the BSA standard. A spectrophotometer (Multiskan GO; Thermo Fisher Scientific, Waltham, MA, USA) was then used for measuring the absorbance at 595 nm (A_595_).

#### TYR inhibitory activity assay

An approach proposed by Batubara et al.^64^. with some modification, was used to conduct the TYR inhibitory activity assay, with L-tyrosine and L-DOPA serving as the substrates, and TYR obtained from mushrooms due to its ease of procurement. Initially, 35 µL of the various protein hydrolysate fractions was mixed with 15 µL of mushroom TYR (333 U/mL) prior to 5 min of incubation at 25 °C. The mixtures subsequently underwent treatment using 55 µL of the substrate (12 mM L-DOPA or 2 mM L-tyrosine) prior to incubation for 30 min at 25 °C. To assess the inhibitory effects towards mushroom TYR of the various hydrolysates, a spectrophotometer was used to measure the absorbance at 490 nm, while kojic acid served as the positive control. Equation (1) below was used to calculate the percentage inhibition of TYR activity:

1 $$\frac{{[({\text{Abs}}\;{\text{control}} - {\text{Abs}}\;{\text{ blank}}) - ({\text{Abs}}\;{\text{sample}}--{\text{Abs}}\;{\text{background}})]}}{{({\text{Abs}}\;{\text{control}}--{\text{Abs}}\;{\text{blank}})}} * 100$$

In which Abs control represents control absorbance, Abs sample indicates the absorbance of the sample, while Abs background refers to the absorbance of the background, which is subtracted from the sample reading, and Abs blank indicates deionized water absorbance. Calculation of the IC_50_ value, defined as the bee pollen hydrolysate concentration capable of inhibiting 50% of the total TYR activity, was then carried out.

### TIP purification

#### UF

Samples were separated by molecular weight via UF (Sartoflow® Smart, Sartorius Stedim Biotech, Göttingen, Germany) using a membrane filter (Pall Corporation, USA). Fractionation of the BPPH produced four different fractions using the MWCO (molecular weight cut off) at 10 kDa, 5 kDa, 3 kDa, and 0.65 kDa. The fractions obtained measured < 0.65 kDa, 0.65-3 kDa, 3–5 kDa, 5–10 kDa, and > 10 kDa, and were placed in storage at -20℃ until required for further analysis. Analysis of the resulting BPPH fractions measured the concentration of protein and the IC_50_ value for the inhibition of TYR, with the best performing fraction in each of the five weight categories chosen for further purification.

#### SEC

The subsequent purification stage following UF made use of a size exclusion column (10 × 300 mm) with Superdex 30 Increase (GE Healthcare Bio-Science AB, Uppsala, Sweden). In addition, ӒKTA pure™ chromatography (GE Healthcare Bio-Science AB, Uppsala, Sweden) alongside a connected Fraction Collector F9-R (GE Healthcare Bio-Science AB, Uppsala, Sweden) was employed. For the procedure, the sample (100 mg) was dissolved in 2 mL distilled water before injection into the column for elution using distilled water at the flow rate of 0.5 mL/min. The resulting 3 mL eluted fractions were collected, whereupon the absorbance was measured at 280 nm along with TYR inhibition.

#### RP-HPLC

Following UF, the additional purification of the fractions offering the greatest TYR inhibitory activity was performed by 0.22 μm filter (Whatman, GE, Buckinghamshire, UK). The resulting purified fraction (50 µL) was then introduced to the RP-HPLC system with its C18 column (Shimpak, 250 × 46 mm, Luna 5 μm, Phenomenex, Torrance, CA, USA). Gradient elution produced the eluted peptides via mobile phase A: 0.1% (v/v) trifluoroacetic acid (TFA) in distilled water, and mobile phase B: 0.05% (v/v) TFA in 70% (v/v) acetonitrile (ACN) while maintaining the flow rate at 0.7 mL/min. A six-phase linear gradient was used for elution as follows: 100:0% (v/v) A: B from 0 to 15 min; 90:10% (v/v) A: B at 15 min; 80:20% (v/v) A: B at 20 min; 70:30% (v/v) A: B at 25 min; 60:40% (v/v) A: B at 30 min, and then a return to 100:0% (v/v) A: B at 35 min. The eluent absorbance was measured using a UV detector at 280 nm, so that those fractions exhibiting high and clear peaks could be collected for further concentration and evaluation of the TYR inhibition. Identification of the peptide sequence was then the subsequent step for the fraction exhibiting the best TYR inhibition.

### Peptide identification via LC-Q-TOF-MS/MS

The peptide fraction selected for its superior TYR inhibition following the RP-HPLC analysis was further investigated to ascertain the amino acid sequence and molecular mass, using a Q-TOF mass spectrometer along with an electrospray ionization source mass spectrometer (ESI; model Amazon SL, Bruker, Germany). Calibration of the ESI-QTOF mass spectrometer was performed via the assessment of peptide chains whose mass values lay in the range of 50 to 25,000 m/z. The Q-TOF mass spectrometry produced data which were then further analyzed *via de novo* sequencing, which is based on the differences in mass when comparing pairs of fragment ions in order to allow the calculation of the mass for each of the amino acid residues in the peptide chain. This makes it possible to identify the amino acid residues. Finally, the NCBI database and the BLASTp tool (https://blast.ncbi.nlm.nih.gov/Blast.cgi) were used to investigate the amino acid sequence and to identify the proteins respectively.

### Bioinformatics tools for peptide property annotation

The effective use of TIPs involves modifications which require a comprehensive understanding of the relevant properties. Profile predictions can be acquired through online database sources for any potential TIPs of interest. This allowed the MS/MS peptide sequences to be compared to the findings reported in the NCBI database using Protein BLAST. Furthermore, estimates of peptide hydrophobicity are available through Peptide2 (www.peptide2.com), while data on peptide solubility are found on the Innovagen server (www.innovagen.com/proteomics-tools), and finally the ToxinPred server (crdd.osdd.net/raghava/toxinpred) provides in silico estimates for peptide toxicity, whereby an SVM score below zero indicates non-toxicity.

### Kinetic study

It is necessary to determine the type of TYR inhibitory activity which takes place. This can be achieved through a modified version of an assay described by Feng et al.^39^. The process starts by dissolving the peptide VY-9 in deionized water at various concentrations (0, 0.25, 0.5, and 1 mM). Each VY-9 peptide sample at each concentration (35 µL) was combined 15 µL of mushroom TYR (333 U/mL in 50 mM PBS pH 6.8) prior to 5 min of incubation at 25 °C. Subsequently, various concentrations of substrate were introduced, namely 0–2 mM L-tyrosine in the case of mono-phenolase, and 0–12 mM L-DOPA for di-phenolase. These new mixtures underwent further incubation for 30 min at the same temperature of 25 °C. Upon completion, a spectrophotometer was used to measure the absorbance and determine the inhibitory activity at 490 nm. Lineweaver-Burk plots were then used in calculating Km and Vmax, the kinetic parameters. Km is indicated by the intersection point on the X-axis while Vmax is indicated by the intersection point on the Y-axis in the mode of TYR inhibition under examination. Construction of the plots linked the substrate concentration reciprocal on the X-axis and the absorbance reciprocal (at 490 nm) on the Y-axis, to represent the starting rate for the enzymatic reaction. A secondary plot, in which the Lineweaver-Burk line slopes (Km/Vmax) were plotted against the concentrations of the peptides was used to establish the inhibitor constant (Ki), whereby the X-axis intersection point in this secondary plot indicated the value for Ki.

### Molecular docking

The molecular docking study used AutoDock Vina to investigate the interactions between TYR, the VY-9 peptide, and arbutin. The docking parameters were configured to encompass the active site, including the Cu ion, which is an essential part of the TYR enzyme’s active site, with the center coordinates defined as center_x = -7.401, center_y = -23.552, and center_z = -32.519. The dimensions of the docking grid box were set to 40 Å x 40 Å x 40 Å, providing ample space to accommodate the peptide and allowing for flexible binding conformations. Among the 20 predicted models, the complex with the highest binding affinity (lowest binding score) was selected for detailed analysis. PyMOL was utilized to generate three-dimensional depictions of the VY-9 peptide in complex with the TYR activity center to visualize the docking results. Furthermore, LigPlot^+^ 2.0 was employed to create detailed two-dimensional interaction maps of the docking conformations. The crystal structure of TYR was sourced from the RCSB Protein Data Bank (PDB ID: 2Y9X, available at https://www.rcsb.org/structure/2Y9X).

### Cell viability assay

To perform the assay to establish cell viability, a 96-well plate was used for the seeding of the B16F10 cells at 5 × 10^3^ cells/well. The cells were then left overnight to allow adherence. The VY-9 peptide at varying concentrations was then used to treat the cells for 72 h. MTT was produced as a 2.5 mg/mL solution in PBS whereupon 10 µL of this MTT solution was introduced to each well before incubating the plate for 4 h at 37 °C inside a humid 5% CO_2_ incubator. Following this step, the medium was removed the wells, and 200 µL of DMSO was added in its place. Finally, a microplate reader was employed to measure the absorbance for each of the wells at 540 nm, while arbutin (0.2 mM) served as the positive control. Each test was carried out in triplicate, with the findings presented in the form of mean ± standard deviation.

### Melanin content assay

In order to assess melanin content, culture flasks were used to grow B16F10 cells (1 × 10^5^ cells/flask) for 24 h. Following this incubation period, various concentrations of the VY-9 peptide (i.e., 0.2, 0.4, 0.8, and 1.6 mM) were used to treat the cells for a period of 48 h. Cell harvesting was conducted by trypsinization and the cells were then rinsed twice in cold PBS. The cell pellets were then dissolved in 0.3 mL of 1 N NaOH to produce solutions which were heated to 90 °C for a one-hour period. The lysates were then placed in a centrifuge for 10 min at 3,000 *× g* whereupon a microplate reader was used to assess the melanin quantity at 405 nm. The protein content in the supernatant was evaluated using the Bradford protein assay with the findings presented in the form of a percentage of the control. Arbutin (0.2 mM) served as the positive control. Each trial was carried out in triplicate, with results presented in the form of mean ± standard deviation.

### Cellular TYR assay

In order to assess the rate of oxidation for L-DOPA, culture flasks were used to first grow B16F10 cells (1 × 10^5^ cells/flask) for 24 h. Following this incubation period, various concentrations of the VY-9 peptide (i.e., 0.2, 0.4, 0.8, and 1.6 mM) were used to treat the cells for a period of 48 h. Cell harvesting was conducted by trypsinization and the cells were then rinsed twice in cold PBS. In the next step, PBS buffer (0.2 mL, pH 6.8) which contained 1% (w/v) Triton X-100 and 1 mM PMSF was introduced to the lysed cell pellets, whereupon amounts of 100 µL of the lysed cells were added to a 96-well plate by pipette, along with 100 µL of 2 mM L-DOPA. This was followed by a one-hour incubation period while the temperature was maintained at 37 °C. A microplate reader was then used to detect the formation of dopachrome in the mixture at 490 nm. The findings are presented in terms of percentage of the control, while arbutin (0.2 mM) served as the positive control. Each test was carried out in triplicate, with the findings presented in the form of mean ± standard deviation.

### RNA isolation and qRT-PCR (quantitative real-time reverse transcription-polymerase chain reaction)

A 6-well plate was used to seed B16F10 cells at 1 × 10^5^ cells per well prior to overnight incubation in a 5% CO_2_ humid atmosphere at 37°C. Fresh medium containing peptides at different concentrations (0.2, 0.4, 0.8, and 1.6 mM) was used to replace the original culture medium, whereupon the cells underwent further culturing for 48 hours. Harvesting was conducted *via* removal of the supernatant, and the cells were rinsed twice in PBS (phosphate-buffered saline). The PureLink™RNA Mini Kit (Invitrogen™, USA) was then used to extract total RNA in line with the guidance of the manufacturer. RNA concentration was assessed at 260 nm with a NanoDrop 2000 UV-Vis spectrophotometer (Thermo Fisher Scientific, Inc.). Oligo-dT primers and a Precision nanoScript II Reverse Transcription Kit (PrimerDesign, Camberley, UK) were used to perform the reverse transcription of 1 µg of total RNA to cDNA in line with the guidance of the manufacturer. MyGo Pro^®^Real time PCR apparatus (IT-IS International Ltd., Stokesley, UK) was then used to perform the qRT-PCR assays in which 20 µL of PCR reaction contained 1 µL of cDNA, 7 µL of ultrapure water, 1 µL of each primer (10 mM), and 10 µL of 2×qPCRBIO SyGreen Mix (PCR Biosystems Ltd, London, UK). This research made use of the following PCR primers: *Gapdh* forward 5’-CTACCCCCAATGTGTCCGTC-3’, *Gapdh* reverse 5’-GCTGTTGAAGTCGCAGGAGAC-3’, *Tyr* forward 5’-CACCGCCCTCTTTTGGAAGT-3’, *Tyr* reverse 5’-AAAGCCTGGATCTGACTCTTGG-3’, *Trp-1* forward 5’-TTCATCTGAGCACCCCTGTCT-3’, *Trp-1* reverse 5’-TTGGCACACTCTCGTGGAAA-3’, *Trp-2* forward 5’-GCTGAACAAGGAATGCTGCC-3’, *Trp-2* reverse 5’-AAGTTTCCTGTGCATTTGCATGT-3’, *Mitf* forward 5’-GGTCTCTGCTCGCCTGATCT − 3’, *Mitf* reverse 5’-GTGATGGTACCGTCCGTGAGAT-3’. The initial stage of the process of qRT-PCR amplification was the activation step of thermal treatment where a temperature of 95 °C was used for 2 min, with 45 subsequent cycles comprising 10 s at 95 °C, 20 s at 60 °C, and 30 s at 72 °C, before embarking upon the final melting curve from 60 to 97 °C with 1 min for each step. The relative gene expression level could then be calculated *via* the cycle threshold (Ct) value, while the role of endogenous control for data normalization was occupied by *Gapdh* (the housekeeping gene). To calculate relative gene expression, the formula given below can be employed:

2 $${\text{Relative}}\;{\text{gene}}\;{\text{expression}}={2^{ - DDCt}}$$

where the heightened threshold cycle for the target gene is given as ΔΔCt. Under this formula, values below 1 show a decrease in the level of gene expression, a value of 1 suggests it has remained constant, while a value above 1 suggests an increase.

### Western blot analysis of the expression of proteins regulating melanogenesis

Western blot analysis was performed to measure TYR (TYR, TRP-1, TRP-2, and MITF) in B16F10 cells in line with the approach discussed by Yoon et al.^65^. whereby the B16F10 cells underwent treatment for 48 h using various peptide amounts (0.2, 0.4, 0.8 and 0.16 mM) and also with arbutin (0.2 mM). Following this process, the cells were lysed using NP40 Cell Lysis Buffer with protease inhibitor cocktail supplementation. Next, 12% sodium dodecyl sulfate poly-acrylamide (SDS-PAGE) gel electrophoresis was used to extract equal quantities of protein from each sample, with subsequent transfer to PVD (polyvinylidene fluoride) membranes (Millipore, Bedford, MA, USA). The membrane was blocked overnight with 3% skimmed milk (w/v) in TBST with 0.5% Tween 20 at 4 °C and then washed thrice in TBST. The rabbit polyclonal anti-TYR antibody (dilution 1:1000), anti-TRP-1 antibody (dilution 1:1000), anti-TRP-2 antibody and anti-MITF antibody (dilution 1:1000) obtained from Abcam (USA), were used respectively to detect the TYR, TRP-1, TRP-2, and MITF bands. After thrice washing again in TBST, secondary antibodies (dilution 1:5000) were added to the membranes for 1 h of incubation at room temperature before another triplicate washing in TBST. Enhanced chemiluminescence horseradish peroxidase (HRP) substrates (GeneDirex, Taiwan) were then used in line with the guidance of the manufacturer to detect bound antibodies, while β-actin antibody was employed to evaluate the loading control. Gel band detection was performed using ImageJ software (version 1.54), available at https://imagej.net/ij/. The software facilitated the identification and quantification of protein expression levels.

### Acute toxicity and melanin inhibitory assay involving zebrafish embryos

To perform the test of acute toxicity, a total of 20 fertilized zebrafish embryos were collected at around 4 h after fertilization and added to each well in a 12-well plate. These embryos then underwent treatment using different quantities of embryo culture medium (2, 4, 6, 8, and 10 µM), while the negative control was distilled water and the positive control was 3,4-dichloroaniline. Incubation of the plate then took place until 48 h after fertilization at a temperature of 28.5 ± 1 °C. The test solutions were refreshed at 24 h after fertilization to allow the counting and removal of any dead embryos. Following exposure, a stereomicroscope (SZX16, Olympus, Japan) was used to record the embryo morphological effects. Meanwhile, a slightly modified version of a previously described melanin inhibitory assay was carried out^30^. The embryos were then homogenized using a cold lysis buffer (PBS pH 7.4, 1% Triton-X and 1% DMSF). The lysate then underwent 10 min of sonication before being placed in a centrifuge for 5 min at 10,000×*g*. The pellet was then removed from the centrifuge and dissolved in 200 µL of 1 N NaOH for 1 h at a temperature of 85 °C. This sample was subsequently vortexed and placed in the centrifuge for a further 5 min at 10,000×*g*. A microplate reader (Spectramax M5, Molecular Devices, USA) was then employed to determine the supernatant absorbance at 490 nm. Melanin content levels could then by calibrated by using the protein amount, and were reported in the form of a percentage compared to the untreated control. In the melanin inhibitory assay, the positive control used was phenylthiourea (PTU) solution (0.0015%).

### Statistical analysis

The experiments in this study were performed in triplicate, with findings shown in the form of mean ± standard deviation. The data analysis then used IBM SPSS Statistics version 29 to assess any significant differences arising between the sample means. Testing of hypotheses was carried out using one-way ANOVA while the significance level was *P* < 0.05.

## Conclusion

This study highlights the value of the neutrase hydrolysate of bee pollen protein as a peptide capable of suppressing TYR. The peptide was screened and step-by-step purified from protein hydrolysate, followed by delineation of tyrosinase inhibitory mechanisms through kinetic and molecular docking studies. The findings indicate that peptide binding takes place at the TYR active site *via* hydrogen bonds and hydrophobic interactions. Bioinformatic predictions have been validated to support its nontoxicity. Surprisingly, VY-9 successfully reduced the amount of melanin and repressed the expression of critical genes and proteins involved in melanogenesis (MITF, TYR, TRP-1, and TRP-2) in B16F10 cells, with no negative impacts on the cells. An examination on animal testing using a zebrafish model indicated that VY-9 has the ability to inhibit melanin formation, indicating its potential as a powerful element in cosmetic and medical sectors. Nevertheless, more research is needed to demonstrate its practical application in commercial products. These investigations necessitate thorough assessments of long-term toxicity and safety, rigorous testing of formulation stability, and diligent clinical trials to determine both effectiveness and safety in humans.

## Electronic supplementary material

Below is the link to the electronic supplementary material.

Supplementary Material 1

## Data Availability

The authors confirm that the data supporting the findings of this study are available within the article and its Supplementary material. Raw data that support the findings of this study are available from the corresponding author, upon reasonable request.
