# Supplementary material for: Bee pollen peptides as potent tyrosinase inhibitors with anti-melanogenesis effects in murine b16f10 melanoma cells and zebrafish embryos
Source: Sci Rep. 2024 Dec 28;14:30834. doi: 10.1038/s41598-024-81495-8 (PMC11681159; doi:10.1038/s41598-024-81495-8)
Supplement: Supplementary file 1 — Supplementary Material 1 [file 41598_2024_81495_MOESM1_ESM.docx]

**Supplement data**

**S1 Figure** Original blot of protein expression of MITF, TYR, TRP-1, TRP-2, and β-actin with three replications. L: protein ladder, C: control cells, A: arbutin (0.2 mM), and VY-9 peptide concentration at 0.2 (P1), 0.4 (P2), 0.8 (P3), and 1.6 (P4) µM.


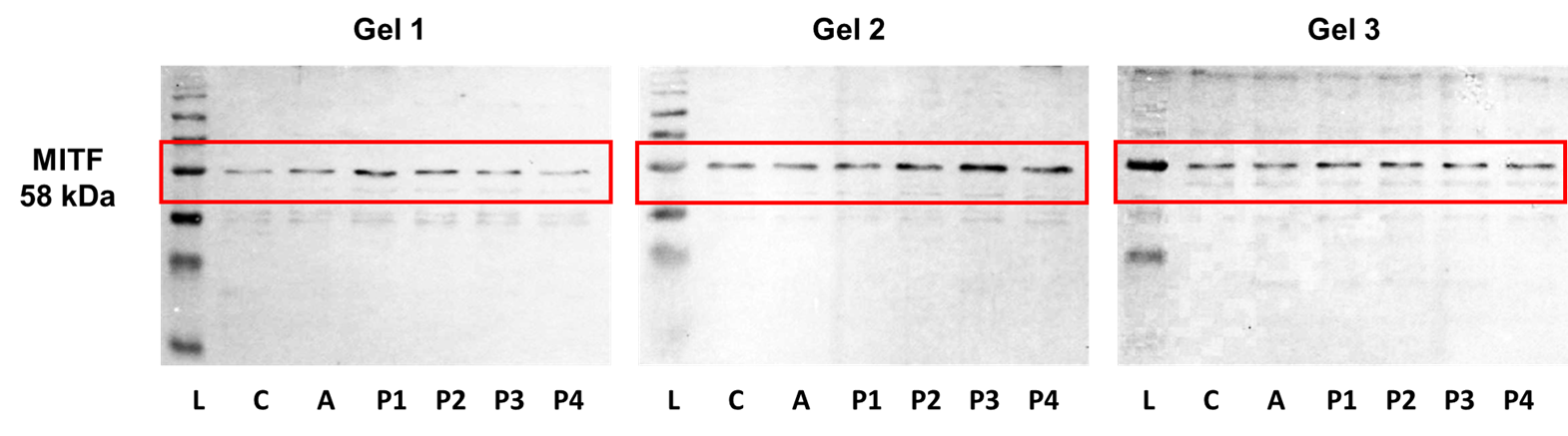


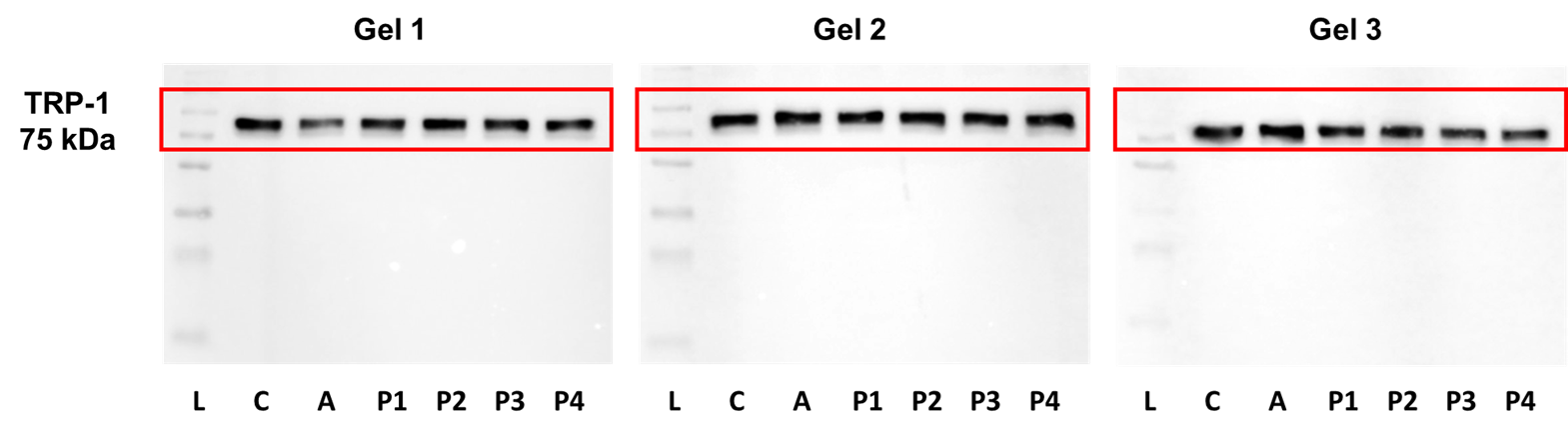

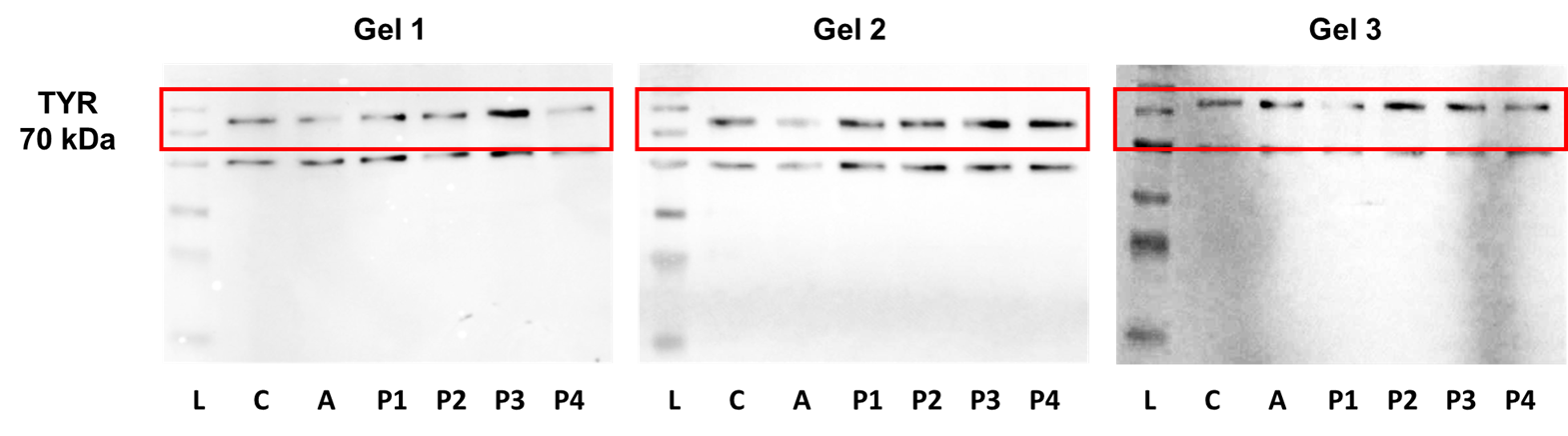


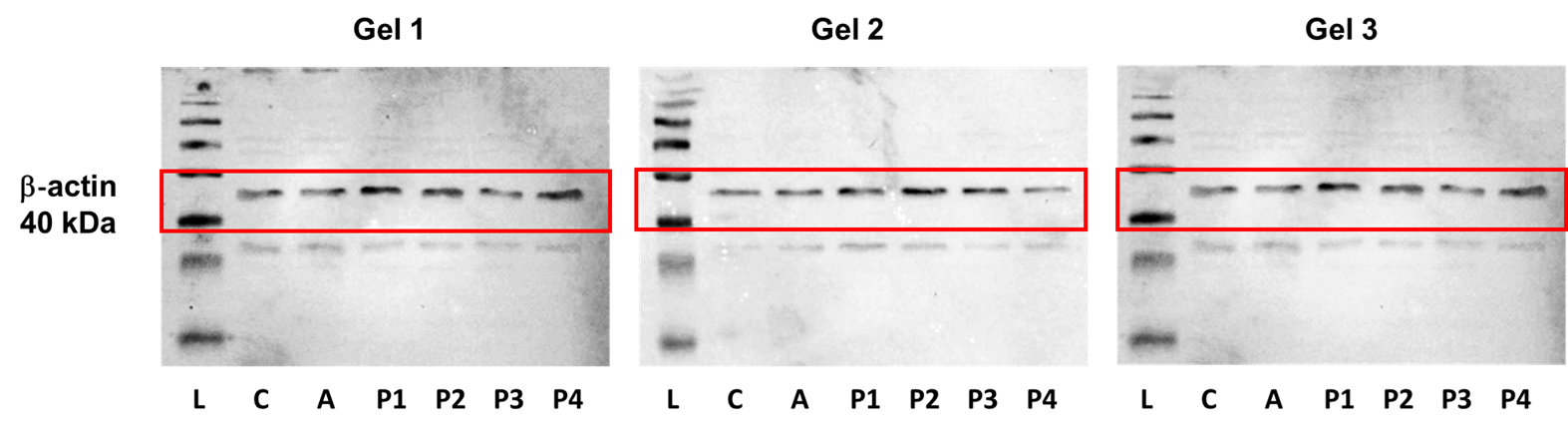

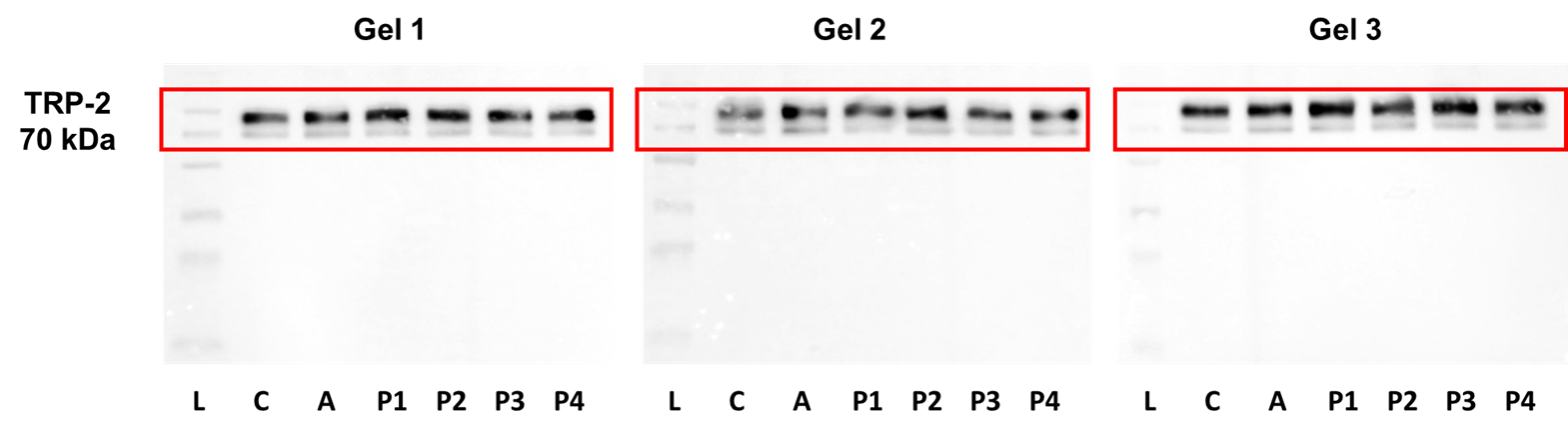


**S2 Figure** The 2D projection of arbutin and TYR docking model. This image was produced using LigPlot.


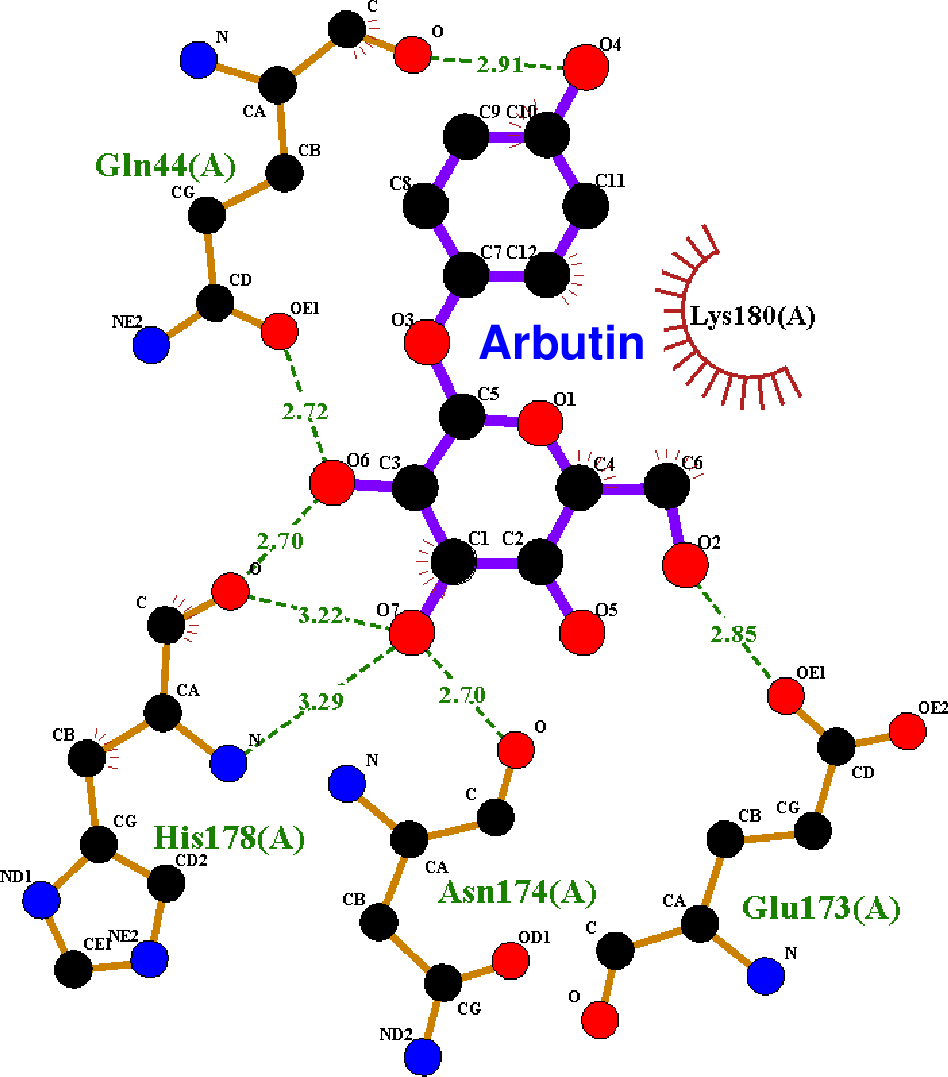


**S1 Table** TYR inhibition activity and IC_50_ values for RP-HPLC Fractions

| **Fractions** | **TYR activity IC_50_ (µg/mL)** | |
| --- | --- | --- |
|  | **Tyrosine (mono-phenolase activity)** | **DOPA (di-phenolase activity)** |
| F_1-1_ | ND | ND |
| F_1-2_ | 17.28±0.02 | 25.69±0.30 |
| F_1-3_ | 33.28±0.55 | 32.65±0.32 |
| F_1-4_ | 32.60±0.48 | 32.18±0.30 |
| F_1-5_ | ND | ND |

ND = Not detected

**S2 Table** Protein BLAST evaluation of the amino acid alignment of the peptide VY-9 peptide in the homologous region.

| **Description (Organism)** |  | **Sequence** | | | | | | | | | | **Accession** |
| --- | --- | --- | --- | --- | --- | --- | --- | --- | --- | --- | --- | --- |
| VY-9 peptide | 1 | **V** | **D** | **G** | **Y** | **P** | **A** | **A** | **G** | **Y** | 9 |  |
| Apyrase (*M. pudica*) | 117 | **V** | **D** | **G** | **Y** | **R** | **V** | **I** | **C** | **G** | 125 | AF062398.1 |
| Ribosomal protein L2 (chloroplast) (*M. tenuiflora*) | 247 | **P** | **W** | **G** | **Y** | **P** | **A** | **L** | **G** | **R** | 255 | QGA84966.1 |
| Ribosomal protein L2 (chloroplast) (*M. bimucronata*) | 248 | **P** | **W** | **G** | **Y** | **P** | **A** | **L** | **G** | **R** | 256 | YP_010315956.1 |
| Ribosomal protein L2 (chloroplast) (*M. pigra*) | 248 | **P** | **W** | **G** | **Y** | **P** | **A** | **L** | **G** | **R** | 256 | YP_010318173.1 |
| Ribosomal protein L2 (chloroplast) (*M. pudica*) | 248 | **P** | **W** | **G** | **Y** | **P** | **A** | **L** | **G** | **R** | 256 | YP_009661880.1 |

**S3 Table** Docking binding energy and interactions over the TYR with arbutin and VY-9 peptide from AutoDock Vina.

| **Ligand** | **Binding energy (kcal/mol)** | | | | |
| --- | --- | --- | --- | --- | --- |
|  | **Mode 1** | **Mode 2** | **Mode 3** | **Mode 4** | **Mode 5** |
| Arbutin | -6.5 | -6.5 | -6.3 | -6.3 | -6.0 |
| VY-9 | -8.3 | -8.2 | -8.1 | -8.0 | -7.9 |
